# Autofluorescence identifies highly phagocytic tissue-resident macrophages in mouse and human skin and cutaneous squamous cell carcinoma

**DOI:** 10.3389/fimmu.2022.903069

**Published:** 2022-10-17

**Authors:** Pierre Bourdely, Luciana Petti, Sokchea Khou, Aida Meghraoui-Kheddar, Roxane Elaldi, Julie Cazareth, Noushine Mossadegh-Keller, Julien Boyer, Michael H. Sieweke, Gilles Poissonnet, Anne Sudaka, Veronique M. Braud, Fabienne Anjuère

**Affiliations:** ^1^ Université Côte d’Azur, CNRS UMR7275, Institut de Pharmacologie Moléculaire et Cellulaire, Valbonne, France; ^2^ Head and Neck Surgery department, Institut Universitaire de la Face et du Cou, Nice, France; ^3^ Université Aix Marseille, CNRS, INSERM, CIML, Marseille, France; ^4^ Center for Regenerative Therapies Dresden (CRTD), Technische Universität Dresden, Dresden, Germany; ^5^ Centre Antoine Lacassagne, Pathology Laboratory, Nice, France

**Keywords:** Macrophages, autofluorescence, function, phagocytosis, skin, squamous cell carcinoma

## Abstract

Macrophages from human and mouse skin share phenotypic and functional features, but remain to be characterized in pathological skin conditions. Skin-resident macrophages are known to derive from embryonic precursors or from adult hematopoiesis. In this report, we investigated the origins, phenotypes and functions of macrophage subsets in mouse and human skin and in cutaneous squamous cell carcinoma (cSCC) using the spectral flow cytometry technology that enables cell autofluorescence to be considered as a full-fledged parameter. Autofluorescence identifies macrophage subsets expressing the CD206 mannose receptor in human peri-tumoral skin and cSCC. In mouse, all AF^+^ macrophages express the CD206 marker, a subset of which also displaying the TIM-4 marker. While TIM-4^-^CD206^+^ AF^+^ macrophages can differentiate from bone-marrow monocytes and infiltrate skin and tumor, TIM-4 identifies exclusively a skin-resident AF^+^ macrophage subset that can derive from prenatal hematopoiesis which is absent in tumor core. In mouse and human, AF^+^ macrophages from perilesional skin and cSCC are highly phagocytic cells contrary to their AF^-^ counterpart, thus identifying autofluorescence as a *bona fide* marker for phagocytosis. Our data bring to light autofluorescence as a functional marker characterizing subsets of phagocytic macrophages in skin and cSCC. Autofluorescence can thus be considered as an attractive marker of function of macrophage subsets in pathological context.

## Introduction

Myeloid cells can be divided into polymorphonuclear cells and cells of the mononuclear phagocyte system (MPS) which encompass dendritic cell (DC) subsets, monocytes and macrophages. Macrophages are found in most adult tissues at steady state and during disease. They are defined as major phagocytic cells with diverse origins and functions depending on tissue location and environmental cues. In tissues, we can identify long-lived tissue-resident macrophages of distinct origins and short-lived macrophages. Except for specific subsets of macrophages that arise from yolk sac precursors during embryonic life, tissue-resident macrophages derive from foetal or adult hematopoiesis including circulating monocytes in steady state (1–3). In addition, during inflammatory processes, some monocytes can be recruited to give rise to short-lived macrophages that disappear during the resolution of inflammation or to long-lived macrophages that persist in the tissue (4).

Tumor-associated macrophages (TAM) represent a prominent population in the microenvironment of progressing solid tumors, where they are often associated with poor clinical outcome, making them major target cells for cancer therapies (5). Recent studies in mouse models identified specialized macrophage subsets with distinct origins contributing differentially to tumor development (6–8). The functional specialization of macrophage subsets in human cancers is still poorly understood. In the era of immunotherapies, refining and identifying markers to assess their functional heterogeneity at steady state and in disease may help to target them more accurately.

Tissue-resident and monocyte-derived macrophages are described in human and mouse skins (9–11). In human skin, MHCII^+^CD11c^+^CD14^+^ populations include both short-lived, monocyte-derived macrophages and long-lived, tissue-resident MHCII^+^CD11c^+^CD14^+^ macrophages known as dermal macrophages. The latter population is highly fluorescent. Analysis of cell intrinsic autofluorescence poses a challenge to conventional flow cytometry as it interferes with other fluorophores (8, 9). The spectral flow cytometry technology based on linear unmixing algorithm resolves this drawback as it allows to analyze autofluorescent spectra as full-fledged parameters (12). Macrophages are enriched in human cutaneous squamous cell carcinoma (cSCC) but their functional heterogeneity remains to be established (13). In the present study, we report the phenotypical and functional heterogeneity of macrophage subsets in mouse and human perilesional skin and cSCC using the spectral flow cytometry technology that enables to manage cell autofluorescence as a full-fledged parameter. We identified autofluorescence as a phagocytic marker that brings to light autofluorescence as a functional marker characterizing subsets of phagocytic macrophages that derive either from prenatal hematopoiesis or from adult monocytes in skin. Autofluorescence can thus be considered as an attractive marker of function of macrophage subsets in pathological context.

## Results

### Distinct subsets of macrophages infiltrate the human skin and cutaneous squamous cell carcinoma

**Figure 1:**
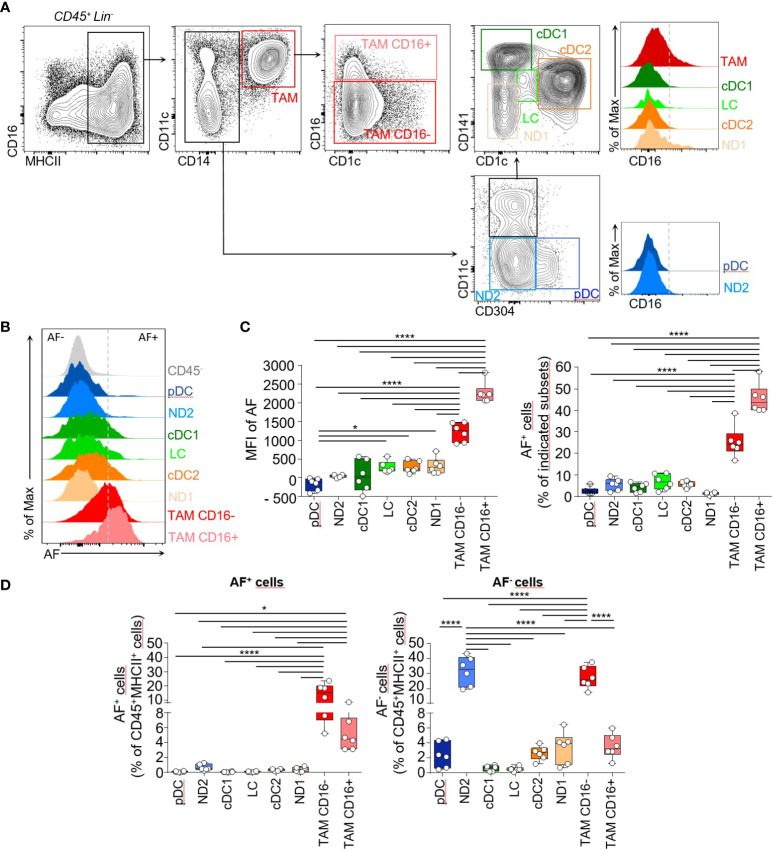
Phenotypic characterization of the myeloid phagocytic system in human cutaneous squamous cell carcinoma identifies a dominant autofluorescent signature in TAM. **(A)** Flow cytometry plots of cell suspensions from human cutaneous squamous cell carcinoma biopsies showing identification of MHCII^+^CD14^+^ tumor-associated macrophages expressing or not CD16 (TAM CD16^-^, red and TAM CD16^+^, pink), MHCII^+^CD14^-^CD11c^-^CD304^+^ plasmacytoid DC (pDC, blue), MHCII^+^CD14^-^CD11c^-^CD304^-^ non-defined cells (ND2), MHCII^+^CD14^-^CD11c^int^CD1c^-^CD141^+^ cDC1 (green), MHCII^+^CD14^-^CD11c^+^CD141^int^CD1c^+^ cDC2 (orange) MHCII^+^CD14^-^CD11c^int^CD141^int^ CD1c^int^ LC (light green) and MHCII^+^CD14^-^CD11c^int^CD1c^-^CD141^-^ non-defined cells (ND1). Histograms showing the CD16 expression by these different cell subsets. Cells were pre-gated on live Lin^-^(CD3^-^, CD19^-^, CD56^-^, CD15^-^) CD45^+^ cells. **(B)** Representative flow cytometry histograms showing autofluorescence intensity for the subsets identified in **(A)**. **(C)** Bar graphs showing the median autofluorescence intensities (left panel) and the frequencies of AF^+^ cells within each indicated cell subset (right panel), n=6. **(D)** Histogram bar graphs showing the frequencies of AF^+^ and AF^-^ cells for the 8 indicated subsets among live Lin^-^CD45^+^MHCII^+^ cells, n=6. **(B-D)** One-way ANOVA, Tukey test, *p<0.05, ****<0.0001.

We used the spectral flow cytometry to characterize the myeloid phagocytic system in human cutaneous squamous cell carcinoma (cSCC) in biopsies from patients that did not receive any anti-inflammatory treatment (**Supplementary Table 1**). The spectral flow cytometry panel included CD45, MHCII (HLA-DR), CD11c, CD14, CD16, CD141, CD1c, CD304 markers, the lineage markers (CD3, CD19, CD56, CD15) and we considered the cell intrinsic autofluorescence (AF) as a full-fledged parameter and the individual fluorescence spectra associated with these tumors were specified (**Supplementary Table 2**; **Supplementary Figures 1A, B** **)**. After gating on live CD45^+^Lineage^-^MHCII^+^ cells, we used CD11c and CD14 to identify CD11c^+^CD14^+^ tumor-associated macrophages (TAM) comprising CD16^+^ TAM and CD16^-^ TAM, CD11c^+^CD14^-^ DC and CD11c^-^CD14^-^ cells. The DC gate encompassed CD141^+^ conventional DC1 (cDC1), CD1c^+^ conventional DC2 (cDC2) and Langerhans cells (LC) expressing intermediate levels of CD1c and CD141 previously identified in human skin (14) as well as a non-defined CD11c^+^CD14^-^ subset (ND1). In the CD14^-^ gate, the CD304 marker identified the plasmacytoid DC subset (pDC) and a non-defined MHCII^+^CD11c^-^CD14^-^CD16^-^CD304^-^ subset (ND2) (**Figure 1A**; **Supplementary Figures 1C, D**). There was heterogeneous expression of autofluorescence (AF) within each above-mentioned subset (**Figure 1B**). CD16^-^ TAM and CD16^+^ TAM were the subsets with the highest autofluorescence (**Figures 1B, C**, left panel) and the highest proportion of AF^+^ cells (**Figure 1C**, right panel). AF^+^CD16^-^ TAM and AF^+^CD16^+^ TAM were also the most abundant AF^+^ cell populations in human cSCC accounting for 14% and 5% of MHCII^+^ cells respectively while DC subsets and non-defined subsets were representing less than 2% of AF^+^ cells altogether among MHCII^+^ cells (**Figure 1D**). This highlights that TAM have the most dominant AF signature in human cSCC. The t-distributed stochastic neighbor embedding analysis (tSNE) of the six concatenated human tumors showing the different subsets identified in **Figure 1A** confirmed the overlap of AF^+^ cells mainly with CD14^+^MHCII^+^ macrophages (**Supplementary Figure 1D**).

**Figure 2:**
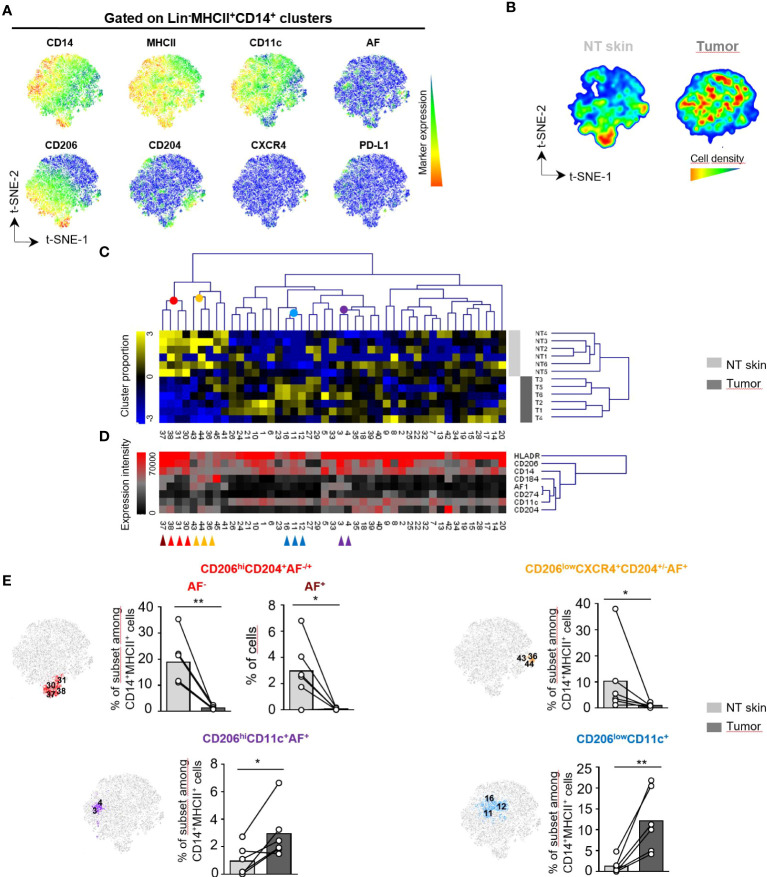
Distinct subsets of macrophages infiltrate human skin and cutaneous squamous cell carcinoma. **(A-E)** Unsupervised analysis of spectral flow cytometry data from human cutaneous squamous cell carcinoma (n=6) and non tumoral (NT) skin (n=6) samples on lin^-^(CD3^-^, CD19^-^, CD15^-^, CD56^-^) CD45^+^MHCII^+^CD14^+^ cells corresponding to macrophage clusters selected from the total population as detailed in **Supplementary Figure 2**. The analysis was done using CD14, MHCII, CD11c, CD206, CD204, CXCR4 and PD-L1 markers as well as cell autofluorescence (AF). **(A)** Macrophage heterogeneity assessed by t-SNE dimensional reduction on live Lin^-^CD45^+^MHCII^+^CD14^+^ cells. t-SNE maps showing expression of CD14, MHCII, CD11c, CD206, CD204, CXCR4, PD-L1 and AF markers on a concatenated file including NT skin and tumor samples from six patients. **(B)** Cell density for the merged file of NT skin and tumor samples shown on a pseudocolor scale. **(C)** Macrophage subsets separated into 45 meta-clusters (MC) by FlowSOM automatic clustering. Heatmap showing the hierarchical clustering of the mean-centered Log2-transformed MC cell proportions for each NT skin (light grey) and tumor sample (dark grey). **(D)** Heatmap representation of Median Fluorescence Intensity (MFI) values of the indicated markers for each MC classified in the same order than in **(C)** Colored arrows identify groups of MC with different proportions in NT skin and tumor samples. **(E)** Three non tumoral skin-enriched MC groups (brown, red and orange arrows shown in **D**) and two tumor-enriched MC groups (blue and violet arrows in **D**) overlaid on a t-SNE-1/t-SNE-2 map and their percentage represented on bar graphs as the cell proportion of each indicated macrophage subset among Lin-CD14^+^MHCII^+^CD45^+^ live cells for each sample. Statistical analysis by paired t test, * p<0.05, ** p<0.01.

AF^+^ tumor-associated macrophages share features with CD14^+^MHCII^+^ dermal macrophages previously described in human skin that have a long lifespan and are highly fluorescent cells when analyzed by conventional cytometry (9, 10). To further phenotype AF^+^ TAM in cSCC, we next evaluated the differential expression of a selected set of macrophage markers (CD14, MHCII, CD11c, CD206, CD204, CXCR4, PD-L1 and AF) in CD45^+^Lin^-^MHCII^+^CD14^+^ cells from tumors and perilesional skin (NT skin) using spectral flow cytometry and computational analysis (**Figure 2**; **Supplementary Table 3**; **Supplementary Figure 2**). Live Lin^-^CD45^+^ subsets were first separated into 10 metaclusters (MC) by FlowSOM automatic clustering in order to select the live Lin^-^CD45^+^CD14^+^MHCII^+^ clusters corresponding to macrophages (**Supplementary Figure 2A**). A t-SNE dimensional reduction analysis then done on selected live Lin^-^CD45^+^CD14^+^MHCII^+^ macrophage subsets shows that skin-associated macrophages were enriched in the lower part of the t-SNE map contrary to tumor-associated ones. In addition, several subsets expressing CD206, CD204 or CXCR4 were overlapping with autofluorescence (**Figures 2A, B**; **Supplementary Figures 2B, C** **)**. Both sample groups had AF^+^ macrophages in similar proportions comprised between 8% and 38% of MHCII^+^ cells (**Supplementary Figure 2C**). To determine the macrophage phenotypes that discriminate cSCC from perilesional skin, we separated the macrophage subsets corresponding to live Lin^-^CD45^+^CD14^+^MHCII^+^ clusters of the first clustering into 45 metaclusters (MC) automatically using the FlowSOM clustering tool. The complete linkage hierarchical clustering and the mean-centered MC cell proportions revealed four groups differentially expressed in tumor and skin (**Figures 2C, D**; **Supplementary Figure 2D**). The first MC group identified CD206^hi^CD204^+^ macrophages as the most abundant subset in perilesional skin, with a cell proportion significantly higher than in tumor (**Figure 2E**, upper left panel; red and brown cells from MC30, MC31, MC37 and MC38 and **Supplementary Figure 2D**). In this MC group, macrophages from MC37 were autofluorescent and may correspond to previously described skin-resident macrophages (10). These cells expressed low levels of CD11c compared to other clusters. The second MC group specifying perilesional skin gathered CD206^low^CD204^+^ CXCR4^+^ cells (**Figure 2E**; orange cells from MC36, MC43 and MC44) that resemble perivascular macrophages identified in tumor periphery (15). These cluster groups that are more abundant in skin than in tumor expressed low levels of CD11c compared to other clusters (CD11c MFI of 2400 and 1855, respectively; range of CD11c intensity for all the MC: 1150-34000). The third MC group characterized CD206^hi^AF^+^ macrophages significantly more abundant in tumor compared to perilesional skin (**Figure 2E**; purple cells from MC3 and MC4 and **Supplementary Figure 2D**). These macrophages differed from perilesional CD206^hi^AF^+^ macrophages (brown cells) in the expression of CD11c and the lack of expression of CD204 marker. The fourth MC group identified a CD206^low^CD11c^+^ macrophage subset enriched in tumor (**Figure 2E**; blue cells from MC11, MC12 and MC16).

Altogether, these data identified distinct subsets of macrophages among which autofluorescent macrophages expressing the CD206 receptor infiltrating human perilesional skin and cSCC.

### Autofluorescent macrophages are enriched in the perilesional skin of mouse TC-1 tumors

To deeply characterize the AF^+^ macrophages from cutaneous squamous cell carcinoma (cSCC) and perilesional skin, we set up a mouse model of cSCC using the syngeneic TC-1 tumor epithelial cell line intradermally implanted in C57BL/6J mice. The myeloid infiltrate of TC-1 cutaneous tumor and perilesional skin was first analysed between day 12 and day 24 post-grafting by spectral flow cytometry using a staining with 14 markers targeting myeloid immune cell populations (see **Supplementary Table 4**, **Supplementary Figure 3** and **Supplementary Figure 4** **)**. After gating on live CD45^+^ cells, a supervised analysis on a representative sample revealed the infiltration of perilesional skin by neutrophils, cDC1 and cDC2 subsets, Langerhans cells (LC) and monocyte-derived DC (moDC) as well as MHCII^+^ macrophages and Ly6C^+^MHCII^-^ monocyte-derived cells (**Supplementary Figure 5A**). The abundance of the myeloid populations in perilesional skin during tumor growth was similar to that of the same subsets in naive skin, with MHCII^+^ macrophages being the most prominent population [**Supplementary Figure 5B** and (11)]. In cutaneous tumor, the proportion of MHCII^+^ macrophages decreased during tumor progression while monocyte-derived Ly6C^+^MHCII^-^ macrophages progressively infiltrated tumor. We identified highly fluorescent cells accounting for around 4% of live CD45^+^ cells in skin while being only rare cells in TC-1 skin tumor and draining lymph nodes (**Supplementary Figure 5C** **)**. As in human samples, autofluorescence was mainly associated to MHCII^+^ macrophages in mouse skin and tumor, and not observed in CD11c^+^MHCII^+^EPCAM^+^CD24^+^ LC (**Supplementary Figure 5D** and data not shown).

**Figure 3:**
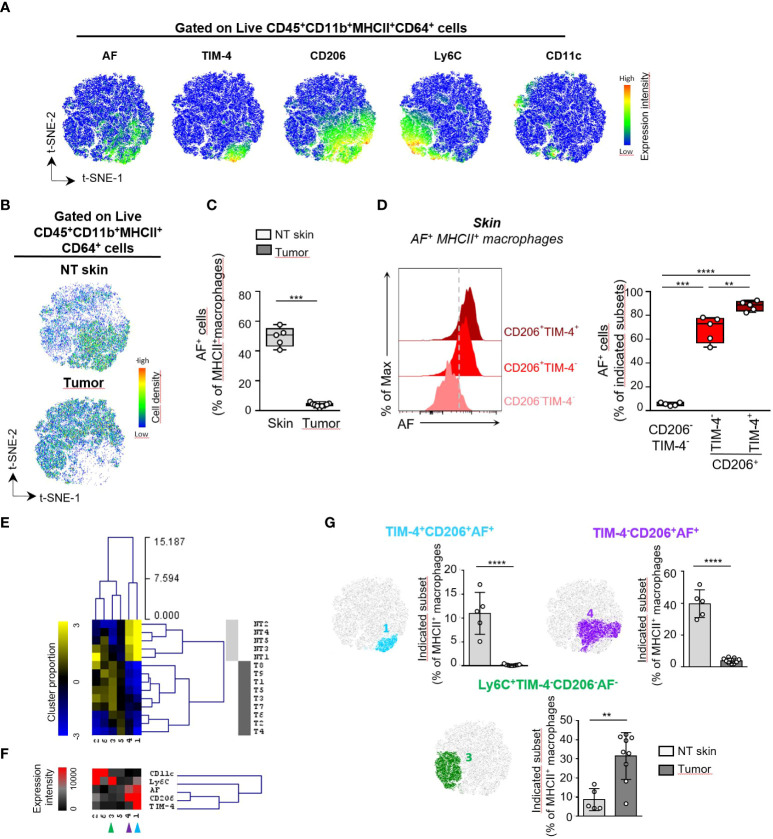
Characterization of MHCII^+^ macrophages based on autofluorescence differentially expressed in mouse skin and TC-1 intradermal tumor. **(A-E)** Unsupervised analysis of spectral flow cytometry data from tumors (n=9) and non tumoral (NT) skin (n=5 pools of skin) of TC-1 intradermal tumor-bearing mice. **(A)** Macrophage heterogeneity assessed by t-SNE dimensional reduction using CD11b, CD64, MHCII, CD11c, Ly6C, CD206, TIM-4 markers and AF parameter among live CD45^+^ CD45^+^CD11b^+^MHCII^+^CD64^+^ cells on a concatenated file including NT skin and tumor samples. **(B)** Pseudocolor density plots for the merged files of NT skin and tumor samples. **(C)** Frequency of AF^+^ macrophages among MHCII^+^ macrophages determined by supervised analysis for NT skin and tumor samples. **(D)** Flow cytometry histograms showing the AF intensity for the indicated skin MHCII^+^ macrophage subsets defined in **Supplementary Figure 6B** and bar graphs showing the frequencies of AF^+^ cells within each indicated cell subset, n=5, paired T test, ** p<0.01, *** p<0.001, **** p<0.0001. **(E)** Macrophages subsets were separated into 6 meta-clusters (MC) by FlowSOM automatic clustering. Heatmap showing the hierarchical clustering of the mean-centered Log2-transformed MC cell proportions for each NT skin (light grey) and tumor (T) sample (dark grey). **(F)** Heatmap representation of Median Fluorescence Intensity (MFI) values of the indicated markers for each MC. **(G)**Two non tumoral skin-enriched MC groups (blue and purple arrows shown in E) and one tumor-enriched MC group (green arrow in F) overlaid on a t-SNE-1/t-SNE-2 map and their cell abundance represented as the cell proportion among live CD45^+^CD11b^+^MHCII^+^CD64^+^ cells for each sample. **(B, F)** Mann-Whitney *U* test, ** p<0.01, *** p<0.001, **** p<0.0001.

To further phenotype mouse AF^+^MHCII^+^ macrophages in skin and TC-1 tumor, we then evaluated the differential expression of a selected set of macrophage markers (CD11b, CD64, MHCII, TIM-4, CD206, Ly6C, CD11c) and autofluorescence in live CD45^+^CD11b^+^MHCII^+^CD64^+^ cells from tumor (T) and perilesional skin (NT skin) using spectral flow cytometry and computational analysis (**Figure 3**, **Supplementary Table 5**, **Supplementary Figure 6**). The t-SNE dimensional reduction using CD11b, CD64, MHCII, CD11c, Ly6C, CD206, TIM-4 markers and AF parameter among gated live CD45^+^CD11b^+^MHCII^+^CD64^+^ cells reveals that AF^+^MHCII^+^ macrophages were distributed on the lower right part of the t-SNE map and overlapped with CD206 and TIM-4 markers with minimal overlap with Ly6C and CD11c markers (**Figure 3A**). These AF^+^MHCII^+^ macrophages were abundant in the perilesional skin but rare cells inside the tumor (**Figures 3B, C**; **Supplementary Figure 6A**). AF^+^ macrophages also infiltrated unpigmented skin from FVB mice (data not shown), thus differentiating them from melanophages previously described in C57BL/6J mouse strain (16).

In mouse skin, CD206 and TIM-4 markers identified three subsets of MHCII^+^ macrophages: a major CD206^+^ subset accounting for 62% of MHCII^+^ macrophages, with around one fourth of this population also expressing the TIM-4 receptor while CD206^-^ macrophages were representing around 32% of MHCII^+^ macrophages and were lacking TIM-4 expression (**Supplementary Figure 6B**). Among these three macrophage subsets, all CD206^+^TIM-4^+^ cells and 70% of CD206^+^TIM-4^-^ cells were AF^+^ macrophages while almost none of the CD206^-^TIM-4^-^ macrophages were autofluorescent (**Figure 3D**). This is consistent with the identification of four main phenotypes: CD206^+^TIM-4^+^ and CD206^+^TIM-4^-^ within AF^+^ macrophages and CD206^-^TIM-4^-^ and CD206^+^TIM-4^-^ macrophages within AF^-^ macrophages (**Supplementary Figure 6C**). To determine the macrophage phenotypes that discriminate tumor from perilesional skin, we then separated the MHCII^+^ macrophages into 6 metaclusters (MC) using the FlowSOM clustering tool. The complete linkage hierarchical clustering and the mean-centered MC cell proportions revealed 4 MC differentially expressed in tumor and skin (**Figures 3E-G**; **Supplementary Figures 6D-E**). Mouse perilesional AF^+^ macrophages corresponding to MC1 and MC4 populations expressed the CD206 marker, consistent with the phenotype observed in AF^+^ macrophages in human skin samples. MC1 corresponded to AF^+^ macrophages expressing the CD206 marker as well as the TIM-4 protein, a marker associated to tissue-resident macrophages (8, 17), suggesting their tissue residency and prenatal origin. CD206^+^TIM-4^+^ macrophages were restricted to non-tumoral skin (**Figure 3F**; **Supplementary Figure 6**). CD206^+^TIM-4^+^ macrophages were exclusively autofluorescent cells characterized by the highest autofluorescence (**Figure 3D**). The MC4 corresponding to CD206^+^TIM-4^-^ AF^+^ macrophages was also significantly more abundant in perilesional skin than in tumor. On the contrary, MC3 corresponded to Ly6C^+^ AF^-^ MHCII^+^ macrophages representing 10% and 38% of the MHCII^+^ macrophages infiltrating the skin and the tumor, respectively. MC3, corresponding to monocyte-derived AF^-^ macrophages, was significantly more abundant in tumor than in skin (**Figure 3F**; **Supplementary Figure 6**).

Consistent with the phenotypes of AF^+^ macrophages identified in human skin and cSCC, mouse AF^+^ macrophages expressed the CD206 receptor and mainly infiltrated perilesional the mouse skin but not the tumor. A CD206^+^ macrophage subset exclusively autofluorescent was characterized by the highest autofluorescence and the expression of the TIM-4 receptor.

### Autofluorescent macrophages comprise subsets of distinct hematopoietic origin

**Figure 4:**
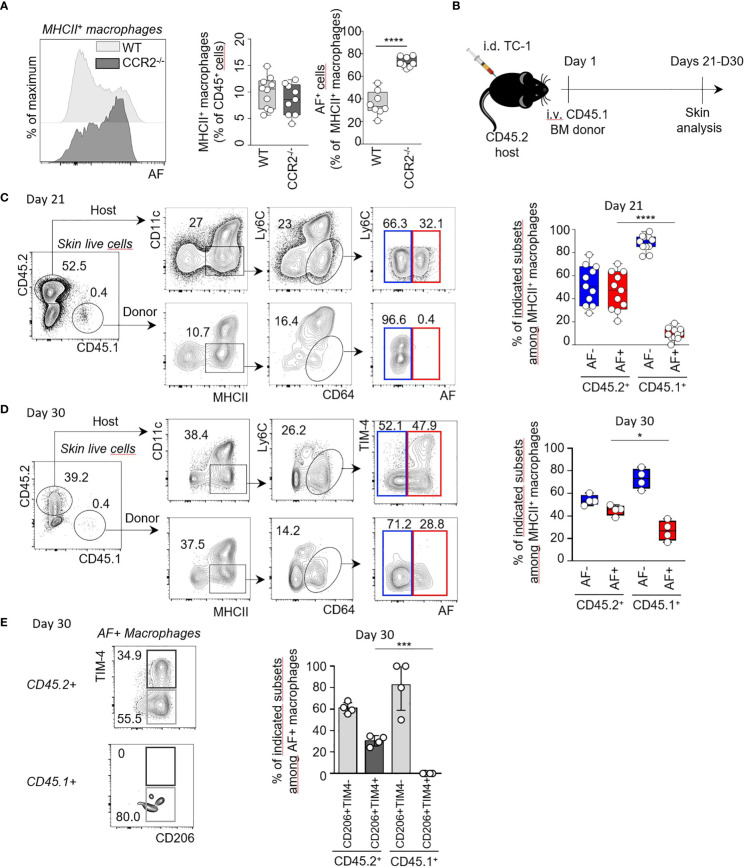
TIM-4^+^ AF^+^ macrophages can derive from prenatal hematopoiesis contrary to their TIM-4- AF^+^ counterpart. **(A)** Representative flow cytometry histograms showing the AF in MHCII^+^ skin macrophages from CCR2^-/-^ and littermate controls (left panel). Quantification of total and AF^+^ macrophages from two independent experiments (n=8 per condition) (right panel). ****p<0.0001, paired t-test. **(B)** Experimental model. CD45.2 mice (host) were injected intra-dermally (i.d.) with 10^4^ TC-1 cells. At Day 1, 10^6^ CD45.1 bone marrow (BM) cells (donor) were injected intravenously (i.v.). Skins from the host mice were collected and analysed by flow cytometry at Day 21 and Day 30. **(C, D)** Representative flow cytometry plots and quantification, at Day 21 and Day 30 showing the frequencies of AF^-^ (blue gate) and AF^+^ (red gate) macrophages among MHCII^+^ macrophages of CD45.2^+^ host or CD45.1^+^ donor cells, respectively. *p<0.05 ****p<0.0001, Mann-Whitney *U* test. **(E)** Representative flow cytometry plots showing TIM-4 and CD206 expression by AF^+^ macrophages of host (CD45.2^+^) or donor (CD45.1^+^) cells. Proportion of CD206^+^TIM-4^-^ and CD206^+^ TIM-4^+^ subsets among AF^+^ macrophages in CD45.2^+^ host and CD45.1^+^ donor cells respectively, at Day 30. ***p<0.001.

To assess the origin of AF^+^ MHCII^+^ macrophages, we characterized the macrophages from perilesional skin using different strains of genetically modified mice. As some skin macrophages differentiate from circulating monocytes which are dependent on the bone marrow egress of CCR2^+^ monocytes (11, 18), we first assessed which proportions of skin AF^+^ macrophages were dependent on CCR2 expression using CCR2^-/-^ mice and control littermates (**Supplementary Table 6**). The proportion of total MHCII^+^ macrophages was not significantly reduced in skin from CCR2^-/-^ mice compared to WT littermates. Interestingly, the proportion of AF^+^ macrophages among total MHCII^+^ macrophages increased by twofold in CCR2^-/-^ skin compared to WT littermate controls to reach 78% of total MHCII^+^ macrophages (**Figure 4A**). This indicated that AF^+^ skin macrophages were less dependent on circulating monocytes than AF^-^ macrophages. We then used non-irradiated recipients to specifically address the developmental potential of AF^+^ macrophages during steady-state conditions.The recipient mice were grafted with cutaneous TC-1 tumor cells at the same time to recapitulate the influence of the TME (**Figure 4B**). We determined the relative abundance of AF^+^ and AF^-^ MHCII^+^ macrophages in the skin of CD45.2^+^ host cells (**Figure 4C**, top panel) and of CD45.1^+^ donor cells (**Figure 4C**, bottom panel 21 days after CD45.1^+^ BM transfer by spectral flow cytometry (**Supplementary Table 7A**). Half of the resident CD45.2^+^ MHCII^+^ cells were AF^+^ macrophages while only rare donor CD45.1^+^ MHCII^+^ macrophages were autofluorescent 21 days after BM grafting contrary to their AF^-^ counterpart (**Figure 4C**, right panel). Nevertheless, it cannot be excluded that AF^+^ macrophages need more than 21 days to differentiate in the skin from BM monocytes. We then assessed the expression of CD206 and TIM-4 by AF^+^ skin macrophages from donor and host mice 21 days and 30 days following transfer of CD45.1 BM cells in CD45.2 recipient mice (**Figures 4D, E**; **Supplementary Table 7B**). Only rare AF^+^ macrophages were of donor origin 21 days after grafting, but their frequency was increasing to represent 26% of CD45.1^+^ macrophages 30 days after grafting (**Figure 4D** and data not shown). CD45.1^+^ AF^+^ macrophages expressed the CD206 but not the TIM-4 marker 30 days after grafting (**Figure 4E**), suggesting that CD206^+^TIM-4^-^ AF^+^ macrophages can derive from post-natal hematopoiesis, mainly from bone marrow monocytes in our experimental conditions, contrary to CD206^+^TIM-4^+^ AF^+^ macrophages. Even if skin AF^+^ macrophages are less dependent on CCR2^+^ monocytes than AF- macrophages (**Figure 4A**), CD206^+^TIM-4^-^ AF^+^ macrophages can differentiate from bone marrow monocytes but need a longer time than their AF^-^ counterpart. On the contrary, skin CD206^+^TIM-4^+^ AF^+^ macrophages were not reconstituted from BM cells in adult mouse in 30 days (**Figure 4E**), which supports the hypothesis that CD206^+^TIM-4^+^ AF^+^ macrophages can be long-lived skin-resident macrophages deriving from prenatal hematopoiesis. To assess the prenatal origin of TIM-4^+^ MHCII^+^ macrophages, a fate mapping experiment in which *Cx3cr1-R26* ^YFP^ embryos E16.5 were pulse-labelled was performed (**Supplementary Figure 7**). This experiment revealed that 90% of TIM-4^+^ MHCII^+^ macrophages expressed the YFP marker as control yolk sac derived microglia cells (**Supplementary Figure 7**). The expression of autofluorescence by TIM-4^+^ macrophages was not assessed in this experiment, but as TIM-4 expression was shown to be restricted to a subset of AF^+^ macrophages present in the skin (**Figures 3F**, **4E**), this suggests that TIM-4^+^AF^+^ macrophages were mainly skin-resident macrophages of prenatal origin.

Altogether, these data indicate that skin AF^+^ macrophages, all expressing the CD206 marker, can have distinct hematopoietic origins. While CD206^+^TIM-4^-^ AF^+^ macrophages can differentiate from bone marrow monocytes, CD206^+^TIM-4^+^ macrophages correspond to skin-resident macrophages mainly derived from prenatal hematopoiesis.

### Autofluorescent macrophages are highly phagocytic cells in mouse and human skin and cSCC

**Figure 5:**
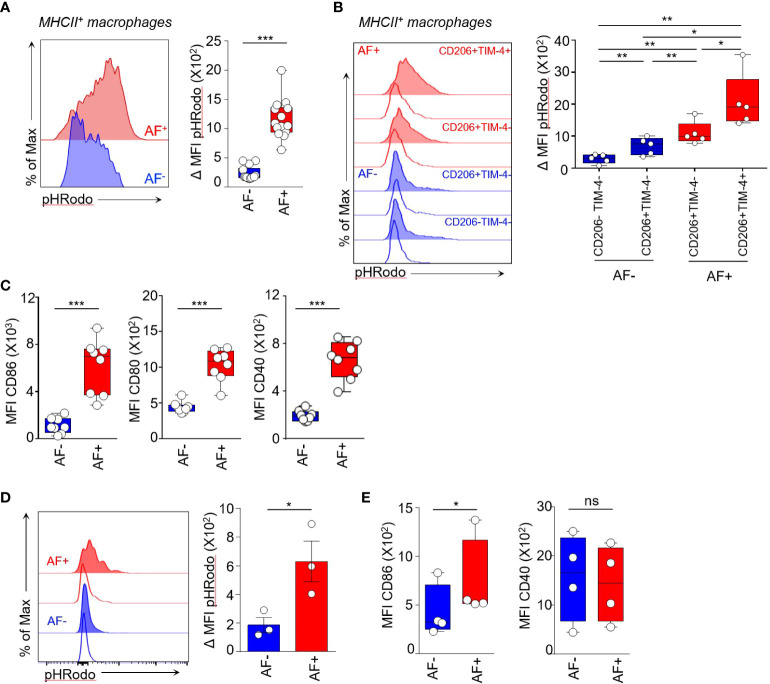
Autofluorescent macrophages are highly phagocytic cells. **(A-C)** Functional assays were performed on NT mouse skin. **(A)** Phagocytic activity of AF^+^ and AF^-^ MHCII^+^ macrophages measured by the uptake of pHrodo green *E.Coli* BioParticle conjugates. Median ΔMFI corresponds to the Median Fluorescence Intensity (MFI) at 37°C minus the MFI at 4°C. Mann-Whitney *U* test, *** p<0.001. **(B)** Histogram representation of pHRodo MFI at 4°C (empty curves) and at 37°C (tinted curves) showing the phagocytic activity of AF^+^ (red) and AF^-^ (blue) macrophage subsets expressing TIM-4 and CD206 markers, only CD206 or double negative for both markers. Bar graphs showing the quantification of phagocytic activity by the indicated macrophage subsets. Median ΔMFI corresponds to the Median Fluorescence Intensity (MFI) at 37°C minus the MFI at 4°C. Paired t-test, *p <0.05 and **p <0.01. **(C)** Expression of Median Fluorescence Intensities (MFI) of CD86, CD80 and CD40 maturation markers by AF^+^ and AF^-^ MHCII^+^ macrophages from skin cell suspensions without stimulation, (n=7), Mann-Whitney *U* test, *** p<0.001. **(D, E)** Functional assays performed on human cutaneous squamous cell carcinoma. **(D)** Histogram representation of pHRodo MFI at 4°C (empty curves) and 37°C (tinted curves) and quantification of phagocytic activity of AF^+^ and AF^-^ TAM measured by the uptake of pHrodo green *E.Coli* BioParticle conjugates. ΔMFI corresponds to the Median Fluorescence Intensity (MFI) at 37°C minus the MFI at 4°C. Paired t-test, *p <0.05. **(E)** Expression of CD86 and CD40 costimulatory molecules by AF^+^ and AF^-^ TAM analyzed by spectral flow cytometry, Paired t-test, *p <0.05. ns, not statistical.

To identify the functions of the AF^+^ macrophages from mouse perilesional skin, we assessed the phagocytic activity of skin cell suspensions *in vitro*, testing their capacity to uptake green fluorescent pHRodo-*E. coli* bioparticles. AF^+^ MHCII^+^ macrophages exhibited the highest phagocytic activity for such bacterial compound as compared to AF^-^ macrophages (**Figure 5A**; **Supplementary Table 8A**). All AF^+^ macrophages also expressed the CD206 receptor (**Figure 3D**; **Supplementary Figure 6C**), a phenotypic marker identifying phagocytic macrophages in intestine and bone marrow (19). AF^+^ macrophages were also endowed with high endocytic capacity contrary to AF^-^ macrophages as shown by the uptake of fluorescent DQ-OVA molecules (**Supplementary Figure 8A**; **Supplementary Table 9**), which is consistent with their dominant expression of CD206, an endocytic receptor involved in the uptake of soluble ovalbumine by antigen-presenting cells (20). Of note, the abundance of AF^+^ macrophages was not modified by such phagocytic assays, showing that autofluorescence is a stable attribute that identifies specific macrophage subsets rather than a characteristics modulated by experimental conditions (**Supplementary Figure 9A** and data not shown).

We then compared the phagocytic activity of CD206^+^TIM-4^+^AF^+^ macrophages and CD206^+^ TIM-4^-^ AF^+^ macrophages testing their capacity to uptake green fluorescent pHRodo-*E. coli* bioparticles (**Figure 5B**; **Supplementary Table 8B**). CD206^+^ TIM-4^+^ macrophages were the highest phagocytic subset (**Figure 5B**). They also were the macrophage subset with the highest autofluorescence (**Figure 3C**) and were accounting for around 16% of MHCII^+^ macrophages (range between 9% and 24% between skin samples) (**Supplementary Figure 8B**). CD206^+^TIM-4^-^ AF^+^ macrophages had significantly lower phagocytic activity compared to CD206^+^TIM-4^-^ AF^+^ macrophages while CD206^+^TIM-4^-^ and CD206^-^TIM-4^-^ AF^-^ macrophages had low phagocytic activity (**Figure 5B**). Thus, these data identified autofluorescence as a functional marker for the phagocytosis of *E coli* bacterial compound that distinguish highly phagocytic CD206^+^TIM-4^+^ macrophages and CD206^+^ TIM-4^-^ macrophages endowed with intermediate phagocytic capacity from AF^-^ macrophages exhibiting low phagocytosis.

AF^+^ macrophages also expressed significantly higher amount of CD86, CD80 and CD40 maturation molecules than AF^-^ MHCII^+^ macrophages in unstimulated conditions, indicating they are more mature cells than AF^-^ macrophages (**Figure 5C**; **Supplementary Table 10**). Furthermore, the expression of CD86 and CD80 protein induced upon *in vitro* stimulation through TLR4 was higher in AF^+^ macrophages than in AF^-^ macrophages (**Supplementary Figure 8C**). As macrophages are also cytokine producers, we compared the expression of TNF-α and IL-6 by skin AF^+^ and AF^-^ macrophages at baseline and upon *in vitro* stimulation (**Supplementary Figures 8D-G**). Notably, the frequencies of AF^+^ and AF^-^ macrophages were not modulated by *in vitro* stimulation and by the use of the exocytosis blockers required to quantify intracellular cytokine expression (**Supplementary Figure 9B**). The proportion of TNF-α^+^ AF^+^ macrophages was significantly the highest as compared to AF^-^ macrophages (36.5% and 20.5%, respectively) upon TLR4 engagement while both skin macrophage populations were low TNF producers in basal conditions (**Supplementary Figures 8D, E**; **Supplementary Table 11**). AF^+^ macrophages had also the highest proportion of IL-6 expressing cells both upon TLR9 engagement and in unstimulatory conditions (**Supplementary Figures 8F, G**, **Supplementary Table 12** **)**. Consequently, autofluorescence discriminated two subsets of MHCII^+^ macrophages with distinct phagocytic capabilities. AF^+^ macrophages are highly phagocytic effectors suggesting that they play a role in preserving tissue homeostasis and in amplifying tissue-associated adaptive immunity. On the contrary, AF^-^ macrophages express low levels of maturation markers and are weak phagocytes.

To check the relevance of our findings in human, we then assessed the phagocytic activity of AF^+^ macrophages in human cSCC. Consistent with mouse data, AF^+^ macrophages had the highest phagocytic activity as compared to their AF^-^ counterpart as shown by their capacity to uptake green fluorescent pHRodo-*E. coli* bioparticles (**Figure 5D**; **Supplementary Table 13**). They also expressed higher levels of the costimulatory molecule CD86 (**Figure 5E**). Thus, AF^+^ human cSCC-associated macrophages are highly phagocytic cells suggesting they represent the tumor-associated equivalent of skin AF^+^ dermal macrophages.

## Discussion

Although the phenotypic and functional diversity of macrophages is recognized in normal and pathological tissues, the functions of distinct subsets are difficult to evaluate *in vivo*. In this study, we have identified that intrinsic cell autofluorescence is a stable marker in specific macrophage subsets from cutaneous squamous cell carcinoma and perilesional skin in mouse and human. Autofluorescence identified highly phagocytic and endocytic MHCII^+^ macrophages and thus can be considered as a marker of cellular function.

All the AF^+^ macrophages express the CD206 mannose receptor, a subset of which also displaying the TIM-4 marker in mouse skin, but CD206 is not restricted to AF^+^ macrophages as a minor subset of AF^-^ macrophages also express CD206 mannose receptor. This indicates that autofluorescence is a more exclusive phagocytic marker than CD206 in the skin environment. AF^+^ macrophage subsets have high phagocytic and endocytic characteristics compared to AF^-^ macrophages, suggesting they can play a role in preserving tissue homeostasis and in amplifying tissue-associated adaptive immunity. Among AF^+^ macrophages, CD206^+^TIM-4^+^ AF^+^ macrophages represent a specific subset characterized by the brightest autofluorescence and the highest phagocytosis capability but absent in tumor core. They can correspond to tissue-resident macrophages derived from prenatal hematopoiesis. Skin CD206^+^TIM-4^-^ AF^+^ macrophages, that infiltrated skin and tumor, are also endowed with phagocytic activity and can differentiate from adult bone marrow monocytes. On the contrary, AF^-^ cells comprise CD206^+/-^TIM-4^-^ MHCII^+^ macrophages, they are CCR2-dependent and are weak phagocytic and endocytic cells. As they massively infiltrate skin tumors, further understanding of their molecular signature in particular in the TME of skin tumors could be relevant to understand their role in tumor progression. These cells may represent monocyte-derived inflammatory macrophages that can be locally reprogrammed to exert cytotoxic effector functions as was shown for recruited TAM in a mouse model of pancreatic tumor (21). This is consistent with their presence in tumors.

Spectral Flow cytometry technology offers the possibility to characterize intrinsic cell autofluorescence in cell suspensions that allows to analyze autofluorescent spectra and to identify them as full-fledged parameters with the use of linear unmixing algorithm. This technology was recently used in a mouse model of asthma to identify multiple cell autofluorescences in lung cells and to substract them to avoid any interference with the phenotypes of immune and non-immune cells (22). In the present report, a main cell autofluorescence was identified in skin and cSCC cells and was managed as a full-fledged cell parameter after linear unmixing, like any other fluorophore. This autofluorescence was shown to be mainly associated to specific subsets of macrophages both in mouse and human while other myeloid cells including conventional dendritic cells and Langerhans cells, specialized in antigen uptake, were not autofluorescent. Consequently, this emerging spectral flow cytometry technology offers the unique possibility to use intrinsic cell autofluorescences as new markers of cell identity.

In adult life, macrophages are innate effectors that reside in most tissues. They can originate from foetal precursors or adult hematopoeisis. In mouse skin at steady state, tissue-resident dermal macrophages can derive either from foetal hematopoiesis or from adult BM-derived progenitors (11). We found that AF^+^ macrophages from mouse skin comprise two subsets, one highly fluorescent that can derive from prenatal hematopoiesis consistent with its expression of the TIM-4 marker, and another MHCII^+^ macrophage subset, less fluorescent and negative for TIM-4 that can differentiate from bone marrow monocytes like AF^-^ macrophages, but in a longer time than non-autofluorescent macrophages. This suggests that intrinsic autofluorescence is a property acquired in the tissue and represent a marker of tissue imprinting rather than of ontogeny. As cell autofluorescence can be due to different molecules that reflect distinct functions, further studies will aim to identify the nature of the autofluorescence of these two macrophage subsets. Identifying the specific autofluorescence spectrum associated to each subset could make these autofluorescences useful phenotypic markers allowing to distinguish AF^+^ tissue-resident dermal macrophages between them and from skin AF^-^ monocyte-derived macrophages in mouse. Highly fluorescent macrophages expressing TIM-4 can be related to a subset of CCR2-independent CD64^+^Ly6C^-^ macrophages characterized in the adult mouse dermis (11). They have a MHCII^+^ mature phenotype and are not replenished from adult BM precursors in 30 days which also supports that these macrophages correspond to long-lived tissue-resident cells. The TIM-4 receptor is restricted to this subset of highly fluorescent macrophages consistent with their prenatal origin as shown for peritoneal macrophages (8, 23). This hypothesis is also supported by the experiment showing that skin TIM-4^+^ macrophages derived from prenatal hematopoiesis using cell-tracing experiments in E16.5 pulse-labelled *Cx3cr1-R26* ^YFP^ progeny.

Consistent with our findings in mouse skin, we reported that autofluorescent and non-autofluorescent macrophage subsets also infiltrate human perilesional skin and cSCC and may relate to tissue-resident and monocyte-derived macrophages, respectively. This is consistent with a previous observation identifying long-lived tissue resident macrophages expressing CD14^+^ and being highly autofluorescent and non-autofluorescent monocyte-derived macrophages in non-pathological human skin (8, 9). Human tumor-associated AF^+^ macrophages, similarly to AF^+^ mouse skin macrophages, expressed higher amount of co-stimulatory molecules than AF^-^ macrophages, underpinning that they are related to tissue-resident dermal macrophages. Like in mouse skin, we reported that human cSCC comprise two subsets of autofluorescent CD14^+^MHCII^+^ macrophages, the one with the highest autofluorescence expressing the CD16 marker. Further studies are needed to definitely determine whether CD16^+^ and CD16^-^ AF^+^ macrophages identified in human perilesional skin and cSCC have different origins or were corresponding to different stages of maturation of a same population. The ontogenic diversity of macrophages in perilesional skin and human cSCC is not surprising as both tissue-resident and monocyte-derived macrophages were already described to populate other tumor types including pancreatic tumors (6), lung tumors (7), serous cavity tumors (8) or brain tumors (24). In these different tumor types, tissue-resident and monocyte-derived macrophages were endowed with distinct functional properties. While tissue-resident macrophages promoted tumor progression in PDAC mouse model through pro-fibrotic properties, monocyte-derived macrophages played a role in tumor antigen sampling (6). In a lung tumor model, tissue-resident macrophages were also associated to tumor progression (7). In contrast, monocyte-derived TAM were associated to tumor progression through pro-fibrotic functions in an adenocarcinoma model (25). Altogether, these studies illustrate the functional heterogeneity of macrophages in the tumor microenvironment and highlight that each tissue exhibits unique specificities that need to be explored in detail. The *in vivo* role of tissue-resident AF^+^ and monocyte-derived AF^-^ macrophages in mouse and human cSCC will be investigated in further studies.

One primary function of macrophages is their phagocytosis activity to eliminate dying cells as well as no pathogenic microbes like yeasts or bacterial pathogens. The mature phenotype, high phagocytic and endocytic characteristics of AF^+^ dermal macrophages and AF^+^ cSCC-associated macrophages in mouse and human suggest they represent a functional subset specialized in tissue repair and antigen uptake. This is highly possible for the TIM-4^+^ AF^+^ macrophage subset identified in mouse skin corresponding to a subset skin-resident macrophages, as TIM-4 is known as a phosphatidyl serine receptor involved in the phagocytosis of apoptotic cells (26). Consistent with our data, a recent study identified phagocytosis as a functional specialization of tissue-resident macrophages in intestine and bone marrow (19). In this study, the CD206 mannose receptor was identified as a bona fide marker of phagocytosis. Both in mouse and human skin environment, highly phagocytic AF^+^ macrophages were characterized by the expression of the CD206 receptor. Nevertheless, in our study, CD206 expression was not restricted to AF^+^ macrophages, suggesting that autofluorescence represents a more exclusive phagocytic marker than CD206 in normal and pathological skin.

In conclusion, our data bring to light autofluorescence as a functional marker characterizing subsets of highly phagocytic macrophages that derive either from prenatal hematopoiesis or from adult monocytes in skin. Consequently, autofluorescence can be used as a phagocytic biomarker in future tissue analyses to further decipher phenotypic and functional characteristics of macrophage subsets in pathological context. Such knowledge will be relevant to design future immunotherapies targeting specialized macrophage subsets in order to block immunosuppression while preserving and stimulating macrophages with antitumoral functions.

## Material and methods

### Patient biopsies

Cutaneous squamous cell carcinoma (cSCC) biopsies were obtained from patients who underwent a surgery at the surgery department of Centre Antoine Lacassagne (Nice, France) in accordance with institutional ethical guidelines. The samples were anonymized and the informed consents of all the patients were collected according to the Declaration of Helsinki with approval of the CAL review board. Patient characteristics are reported in **Supplementary Table 1**.

### Mouse breeding and cutaneous cancer model

Six-week old CD45.2 and CD45.1 C57BL/6J mice were purchased from Charles River Laboratories. Female CCR2^-/-^ mice and control littermates were obtained from in house breeding. CX3CR1^cre-ER:R26-YFP^ mice used for fate mapping experiments, obtained by crossing CXCR3^cre-ER^ and Rosa26^LSL-YFP^ mice from Jackson laboratories, were provided by CIML (Marseille, France). All animal experiments were performed in compliance with institutional and national guidelines and have been approved by the regional committee for animal experimentation. The TC-1 epithelial tumor cell line was generated as described elsewhere (15). TC-1 cells (2x10^4^ cells) were injected intradermally (i.d.) in 10 μL of PBS with an insulin syringe in the shaved dorsal back skin of anesthetized mice. The tumoral volumes were measured using the ellipsoid formula π/6*H*W*D.

### Adoptive bone marrow transfer

Female CD45.2^+^ C57BL/6J mice were grafted intradermally with TC-1 tumor cells as previously described at Day 1 and were injected i.v. with 10.10^6^ bone marrow cells from femurs and tibias of CD45.1^+^ C57BL/6J.

### Fate mapping experiments

Genetic fate-mapping using CX3CR1^cre-ER:R26-YFP^ mice were performed at CIML as previously described (27). Briefly, pregnant females were pulsed labeled at E16.5 by intraperitoneal injection of 0.1 mg/kg tamoxifen and 0.05mg/kg progesterone and 8-week old progeny used for characterization of skin macrophages.

### Cell preparation from mouse and human tissues

Mouse dorsal skins were cut in small pieces and cells dissociated by two successive enzymatic digestion with collagenase IV (0.6 mg/mL) (Sigma-Aldrich) at 37°C for 20 minutes under constant shaking. Mouse TC-1 tumors were cut in small pieces followed by one enzymatic digestion with collagenase IV from Clostridium histolyticum (1mg/mL) (Sigma-Aldrich) and DNAse II from bovine pancreas (0.2 mg/mL) (Sigma-Aldrich) at 37°C for 20 minutes under constant shaking. Skin and tumor cell suspensions were filtered through a 70µm cell strainer to eliminate aggregates. Cell suspensions were enriched in immune cells by 80/37.5% Percoll gradient centrifugation (Amersham GE Healthcare Life Sciences) and used for stainings and *in vitro* stimulations. Brain tissue was macerated and taken up in HBSS containing 0.5% d-glucose (Sigma) and 15 mM Hepes (Life Technologies). The resulting cell suspension was passed through 70µm cell strainers and subjected to a 70/37/30% Percoll gradient, from which microglia cells were isolated. After mechanical dilaceration, human cSCC tumor biopsies and perilesional skins were digested with collagenase IV from Clostridium histolyticum (1.25 mg/mL) (Sigma-Aldrich) and DNAse II-S from bovine pancreas (0.13 mg/mL) (Sigma-Aldrich) at 37°C for 40 minutes under constant shaking. Cell suspensions were filtered through a 70µm cell strainer to eliminate aggregates and used for stinings and *in vitro* stimulations.

### Phagocytic assays

For pHRodo assay, cell suspensions from skins or tumors were first stained with antibodies directed against surface markers as described in flow cytometry section and corresponding supplementary tables (**Supplementary Tables 8**, **13**, for mouse and human samples, respectively). Stained cells were then incubated for 15 min at 37°C or 4°C with 0.5 mg/mL of pHrodo green *E.Coli* bioparticules (Life Technologies), washed, and then analyzed by flow cytometry. Add For DQ-OVA processing assay, skin cell suspensions were incubated for 15 minutes at 37°C with 10 µg/mL DQ-OVA (Molecular Probes) for antigen uptake. Cells were thoroughly washed and then incubated additional times to measure dye processing. After different times of incubation, cells were stained with the panel described in **Supplementary Table 9** and analyzed by spectral flow cytometry.

### *In vitro* stimulation

Mouse and human cell suspensions were incubated with LPS EB-ultrapure from *E. coli* O111:B4 (1 μg/mL, *In vivo*gen) or CpG-ODN 2395 (5μM, *Invivo*gen) at 37°C for 4 hours in presence of Golgi stop and Golgi plug (BD Biosciences) and then assessed for cytokine expression by intracellular staining.

### Flow cytometry and computational analysis

Cell suspensions from mouse skin and tumors were preincubated with purified anti-CD16/CD32 mAb (clone 2.4G2; BD Biosciences) 15 minutes at 4°C to prevent unspecific binding. All antibodies panels (see lists in **Supplementary Tables 4-12**) were prepared in Brilliant Stain Buffer (BD Biosciences) and incubated with cell suspensions for 30 minutes at 4°C. Cell suspensions from human tumors and perilesional skins were preincubated with mouse serum (1/10) (Biowest) and IgG from human serum (100 µg/mL) (Sigma-Aldrich) 30 minutes at 4°C to prevent aspecific binding. All antibodies panels (see lists in **Supplementary Tables 2, 3, 13 and 14**) were prepared in PBS 0.1% bovine serum albumin (Sigma-Aldrich) 5mM EDTA 0.05% sodium azide (Merck) and incubated with cells for 30 minutes at 4°C. Before acquisition, stained cell suspensions were then incubated with the indicated viability dyes according the manufacturer instructions. For intracellular stainings, dead cells were labeled with live/dead yellow (Thermo Fischer), aqua or near infra-red zombie dyes (Biolegend). Cells were then fixed and permeabilized with Cytofix/cytoperm kit (BD Biosciences). Data were acquired on a SP6800 spectral cytometer (SONY Biotechnology) (12) and analyzed with FlowJo V10 software (Treestar) using supervised and unsupervised analytical approaches. For unsupervised analyses, manual gating was done to exclude residual debris, doublets and dead cells. Live cells were gated using FlowJo as previously detailed (28, 29). Dimensional reduction of the data was performed selecting the indicated markers by t-distributed stochastic neighbor embedding (t-SNE) using the FlowJo package. MetaClusters (MC) were generated using the FlowSOM package. The frequency of each MC was represented as a heatmap (Mean-centered, log2 transformation) using MeV software. Median intensity values per cluster for each marker were calculated. They were represented on a heatmap of marker expression in which each MC was ordered based on its position in the hierarchical clustering of cell proportions (**Figure 2C**) to visualize the phenotype of each MC. The identity of each cluster was inferred based on the expression of each individual marker. For the analysis of human samples, a hierarchical clustering including 45MC was selected to efficiently separate skin and tumor samples. In this clustering, some MC were grouped applying the following criteria: a) a statistical comparison of the cell proportion of each MC in NT skin and tumor groups to identify the MC that were differentially expressed in one group; b) these MC clusters were grouped with proximal MC when they were sharing parents within two sub-branch points together with their proximity on the t-TSNE map indicating they were sharing similar phenotype. The level of expression of CD206 marker was labelled high when the median fluorescence intensity of this marker was significantly higher for a cluster than the mean of the MFI of all the clusters for the same marker, and was named as low when the CD206 MFI for a cluster was below the mean of the MFI of all the clusters for the same marker.

### Statistical analysis

Statistical analyses were carried out with Prism software, version 6.0 (GraphPad Prism, San Diego, CA).

## Data availability statement

The raw data supporting the conclusions of this article will be made available by the authors, without undue reservation.

## Ethics statement

The studies involving human participants were reviewed and approved by Antoine Lacassagne Cancer Center (CAL) institutional review board. The patients provided their written informed consent to participate in this study. The animal study was reviewed and approved by the Regional Committee for Animal Experimentation.

## Author contributions

PB and FA conceived the research. PB, LP, JC, SK, AM-K and RE performed experiments and/or analyzed the data. RE, JB, AS, and GP provided human samples and clinical expertise. NM-K and MS contributed to analytical tools and expertise in ontogeny experiments. FA and VB obtained funding. PB, LP, and FA wrote the manuscript. FA supervised the study and revised the submitted manuscript. All authors contributed to the article and approved the submitted version.

## Funding

This study was supported by the following funding agencies: Centre National de la Recherche Scientifique, Institut National de la Santé Et de la Recherche Médicale, Université Côte d’Azur, Région Provence-Alpes-Côte d’Azur, French Government (National Research Agency, ANR) through the “Investments for the Future” programs LABEX SIGNALIFE ANR-11-LABX-0028 and IDEX UCAJedi ANR-15-IDEX-01, INCa/LNCC ECMpact (AAP2017.LNCC), Cancéropole PACA, Fondation ARC pour la recherche sur le Cancer, Fondation de l’Avenir, Ligue Nationale contre le Cancer, Fondation d’entreprise SILAB Jean PAUFIQUE, and Fondation Bristol-Myers Squibb (BMS).

## Acknowledgments

We thank A. Rubod and N. Hippolite for expert technical assistance and Dr. J. Haudebourg as well as all the technical staff of the pathology laboratory of Nice Cancer Center (Centre Antoine Lacassagne, CAL, Nice, France) for the extemporaneous preparation of human biopsies and histological analyses. We thank the IPMC’s Imaging/Flow cytometry and animal house core facilities for providing assistance.

## Conflict of interest

The authors declare that the research was conducted in the absence of any commercial or financial relationships that could be construed as a potential conflict of interest.

## Publisher’s note

All claims expressed in this article are solely those of the authors and do not necessarily represent those of their affiliated organizations, or those of the publisher, the editors and the reviewers. Any product that may be evaluated in this article, or claim that may be made by its manufacturer, is not guaranteed or endorsed by the publisher.
