# Supplementary material for: Autofluorescence identifies highly phagocytic tissue-resident macrophages in mouse and human skin and cutaneous squamous cell carcinoma
Source: Front Immunol. 2022 Oct 17;13:903069. doi: 10.3389/fimmu.2022.903069 (PMC9619110; doi:10.3389/fimmu.2022.903069)
Supplement: Supplementary file 1 [file DataSheet_1.pdf]

AUTOFLUORESCENCE IDENTIFIES HIGHLY PHAGOCYTIC TISSUE-RESIDENT MACROPHAGES IN MOUSE AND HUMAN SKIN AND CUTANEOUS SQUAMOUS CELL CARCINOMA

Pierre Bourdely[1,a§], Luciana Petti[1§], Sokchea Khou[1,b], Aida Meghraoui-Kheddar[1c], Roxane Elaldi[1,2], Julie Cazareth[1], Noushine Mossadegh-Keller[3], Julien Boyer[4], Michael H Sieweke[3,4], Gilles Poissonnet[2], Anne Sudaka[5], Veronique M Braud[1] and Fabienne Anjuère[1]*

Additional information

Supplementary information accompanies this paper (16 supplementary tables and 9 supplementary figures).

Supplementary tables

Supplementary Table 1 : Patients' characteristics

	Characteristics	Number
Gender		
	Male	14 (100%)
Age		
	mean (years ± sem)	78.5±2,2
	range	65-91
History		
	no anti-inflammatory medication	14
	no immunocompromised	12
	no cancer treatment	12
Tumor location		
	temple	3
	scalp	4
	forehead	1
	cheek	1
	external ear	3
	lip	1
	nose	1
Histology		
	undifferentiated cSCC	1
	moderately differentiated cSCC	5
	well differentiated cSCC	8
BWH classification		
	T1	1
	T2a	8
	T2b	4
	T3	1

*Brigham and Women’s Hospital tumor classification system : classification system based on risk factors specific for cSCC (Jambusaria-Pahlajani et al., 2013). Risk factors include tumor diameter of 2 cm or larger, poorly differentiated histology, perineural invasion and tumor invasion beyond the subcutaneous fat (excluding bones which automatically upgrade to T3).

Supplementary Table 2 : Human antibody panel for identification of myeloid subsets

Markers	Fluorochrome	Clone	Provider
CD1c	BV421	F10/ 21A3	BD
Dead cell marker	Zombie Aqua dye		Biolegend
CD3	FITC	UCHT1	BD
CD19	FITC	HIB19	BD
CD56	FITC	B159	BD
CD15	FITC	HI98	BD
CD141	PE	1A4	BD
HLA-DR	PECf594	G46-6	BD
CD45	PercpCy5.5	HI30	BD
CD11c	PeCy7	B-ly6	BD
CD304	APC	U21-1283	BD
CD16	A700	3G8	BD
CD14	APC H7	MOP9	BD

Supplementary Table 3 : Human antibody panel for phenotyping AF^{+} macrophages

Markers	Fluorochrome	Clone	Provider
CD206	BV421	15-2	Biolegend
CD14	BV510	ME52	Biolegend
CD184/CXCR4	BV605	12G5	BD
CD163	BV650	GHI/61	BD
CD274/PD-L1	BV785	29E.2.A.3	Biolegend
CD3	FITC	UCHT1	BD
CD19	FITC	HIB19	BD
CD56	FITC	B159	BD
CD15	FITC	HI98	BD
Dead cell marker	7-AAD		Biolegend
CD45	PerCP-C5.5	HI30	BD
CD204	PE	U23-56	BD
HLA-DR	PE-CF594	G46-6	BD
CD11c	PeCy7	B-ly6	BD

Supplementary Table 4 : Mouse antibody panel for identification of myeloid subsets

Markers	Fluorochrome	Clone	Provider
MHCII	BV421	M5/114	BD
CD45	BV510	30F-11	BD
CD24	BV605	M1/69	BD
CD11c	BV650	N418	Biolegend
CD64	BV711	X54-5/7.1	Biolegend
CD3	BV786	145-2C11	BD
Epcam	BB515	G8.8	BD
Siglec H	FITC	551	Biolegend
Ly6G	PE	1A8	BD
CD103	PE	M290	BD
Dead cell marker	7-AAD		BD
Ly6C	PE-CF594	HK1.4	BD
XCR1	PercpCy5.5	ZET	Biolegend
CD11b	PeCy7	M1/70	BD

Supplementary Table 5: Mouse antibody panels for phenotyping AF^+ skin macrophages

Markers	Fluorochrome	Clone	Provider
MHCII	BV421	M5/114	BD
CD45	BV510	30F-11	BD
CD24	BV605	M1/69	BD
CD11c	BV650	HL3	BD
CD64	BV711	X54-5/7.1	Biolegend
Dead cell marker	7-AAD		BD
EPCAM	BB515	G8.8	BD
CD206	PerCPCy5.5	C068C2	Biolegend
Tim-4	PE	RMT4-54	BD
CD11b	PeCy7	M1/70	BD
Ly6C	PE-CF594	AL621	BD

Supplementary Table 6 : Antibody panel for analysis of AF^+ macrophages in $CCR2^{-/-}$ mice

Markers	Fluorochrome	Clone	Provider
MHCII	BV421	M5/114	BD
CD45	BV510	30F-11	BD
CD64	BV711	X54-5/7.1	Biolegend
CCR2	PE	475301	R&D
Dead cell marker	7-AAD		BD
CD11c	Pe Dazzle594	N418	Biolegend
CD11b	PeCy7	M1/70	BD

Supplementary Table 7a : Mouse antibody panel for CD45.1 transfer experiment

Markers	Fluorochrome	Clone	Provider
MHCII	BV421	M5/114	BD
CD45.2	BV510	104	BD
CD24	BV605	M1/69	BD
CD11c	BV650	N418	Biolegend
CD64	BV711	X54-5/7.1	Biolegend
CD45.1	FITC	A20	BD
Ly6G	PE	1A8	BD
CD103	PE	M290	BD
Dead cell marker	7-AAD		BD
Ly6C	PE-CF594	HK1.4	BD
XCR1	PercpCy5.5	ZET	Biolegend
CD11b	PeCy7	M1/70	BD

Supplementary Table 7b : Mouse antibody panel for CD45.1 transfer experiment

Markers	Fluorochrome	Clone	Provider
MHCII	BV421	M5/114	BD
CD45.2	BV510	104	BD
CD24	BV605	M1/69	BD
CD11c	BV650	HL3	BD
CD64	BV711	X54-5/7.1	Biolegend
Dead cell marker	7-AAD		BD
CD45.1	FITC	RMT4-54	BD
CD206	PerCPCy5.5	C068C2	Biolegend
Tim-4	PE	RMT4-54	BD
CD11b	PeCy7	M1/70	BD
Ly6C	PE-CF594	AL621	BD

Supplementary Table 8a : Mouse antibody panel for phagocytosis analysis

Markers	Fluorochrome	Clone	Provider
MHCII	BV421	M5/114	BD
CD45	BV510	30F-11	BD
CD64	BV711	X54-5/7.1	Biolegend
E Coli bioparticles	PHrodo green		Invitrogen
Dead cell marker	7-AAD		BD
CD11c	Pe Dazzle594	N418	Biolegend
CD11b	PeCy7	M1/70	BD

Supplementary Table 8b : Mouse antibody panel for phagocytosis analysis by TIM-4 and CD206 macrophages

Markers	Fluorochrome	Clone	Provider
MHCII	BV421	M5/114	BD
CD45	BV510	30F-11	BD
CD24	BV605	M1/69	BD
CD11c	BV650	HL3	BD
CD64	BV711	X54-5/7.1	Biolegend
Dead cell marker	7-AAD		BD
E Coli bioparticles	pHRodo green		Invitrogen
CD206	PerCPCy5.5	C068C2	Biolegend
Tim-4	PE	RMT4-54	BD
CD11b	PeCy7	M1/70	BD
Ly6C	PE-CF594	AL621	BD

Supplementary table 9: Mouse antibody panel for DQ-OVA endocytosis and processing

Markers	Fluorochrome	Clone	Provider
MHCII	BV421	M5/114	BD
CD45	BV510	30F-11	BD
CD64	BV711	X54-5/7.1	Biolegend
DQ-OVA			Thermo Fischer
Dead cell marker	7-AAD		BD
CD11c	Pe Dazzle594	N418	Biolegend
CD11b	PeCy7	M1/70	BD

Supplementary Table 10 : Mouse antibody panel for analysis of maturation markers

Markers	Fluorochrome	Clone	Provider
MHCII	BV421	M5/114	BD
CD86	BV510	GL1	BD
CD64	BV711	X54-5/7.1	Biolegend
CD45	BV786	30F-11	BD
CD40	FITC	mars-23	BD
CD80	PE	16-10A1	BD
Dead cell marker	7-AAD		BD
CD11c	Pe Dazzle594	N418	Biolegend
CD11b	PeCy7	M1/70	BD

Supplementary Table 11 : Mouse antibody panel for analysis of TNF-α expression

Markers	Fluorochrome	Clone	Provider
MHCII	BV421	M5/114	BD
CD45	BV510	30F-11	BD
Dead cell marker	Live/dead Yellow dye		Molecular Probes
CD64	BV711	X54-5/7.1	Biolegend
TNFα	PE	MP6-XT22	Ebiosciences
CD11c	Pe Dazzle594	N418	Biolegend
CD11b	PeCy7	M1/70	BD

Supplementary Table 12 : Mouse antibody panel for analysis of IL-6 and IL-10 expression

Markers	Fluorochrome	Clone	Provider
MHCII	BV421	M5/114	BD
Dead cell marker	Zombie Aqua dye		Biolegend
CD45	BV786	30F-11	BD
IL-6	A488	MP5-20F3	BD
IL-10	PE	JES5-16E3	BD
CD11c	Pe Dazzle594	N418	Biolegend
CD11b	PeCy7	M1/70	BD
CD64	A647	X54-5/7.1	Biolegend

Supplementary Table 13 : Human antibody panel for phagocytosis analysis

Markers	Fluorochrome	Clone	Provider
E Coli bioparticles	pHRodo green		Invitrogen
Dead cell marker	7-AAD		Biolegend
HLA-DR	PECf594	G46-6	BD
CD45	PercpCy5.5	HI30	BD
CD204	PE	U23-56	BD
CD11c	PeCy7	B-ly6	BD
CD14	BV510	M5E2	Biolegend

Supplementary Table 14 : Human antibody panel for functional analysis of AF^+ macrophages

Markers	Fluorochrome	Clone	Provider
CD86	BV421	FUN-1	BD
Dead cell marker	Zombie aqua dye		Biolegend
CD3	FITC	UCHT1	BD
CD19	FITC	HIB19	BD
CD56	FITC	B159	BD
CD15	FITC	HI98	BD
TNF-α	PE	MAb11	BD
HLA-DR	PECf594	G46-6	BD
CD45	PercpCy5.5	HI30	BD
CD11c	PeCy7	B-ly6	BD
CD40	APC	rAB89	Beckman coulter
CD14	APC H7	MOP9	BD

Supplementary Figures

Supplementary Figure 1 Bourdely et al.

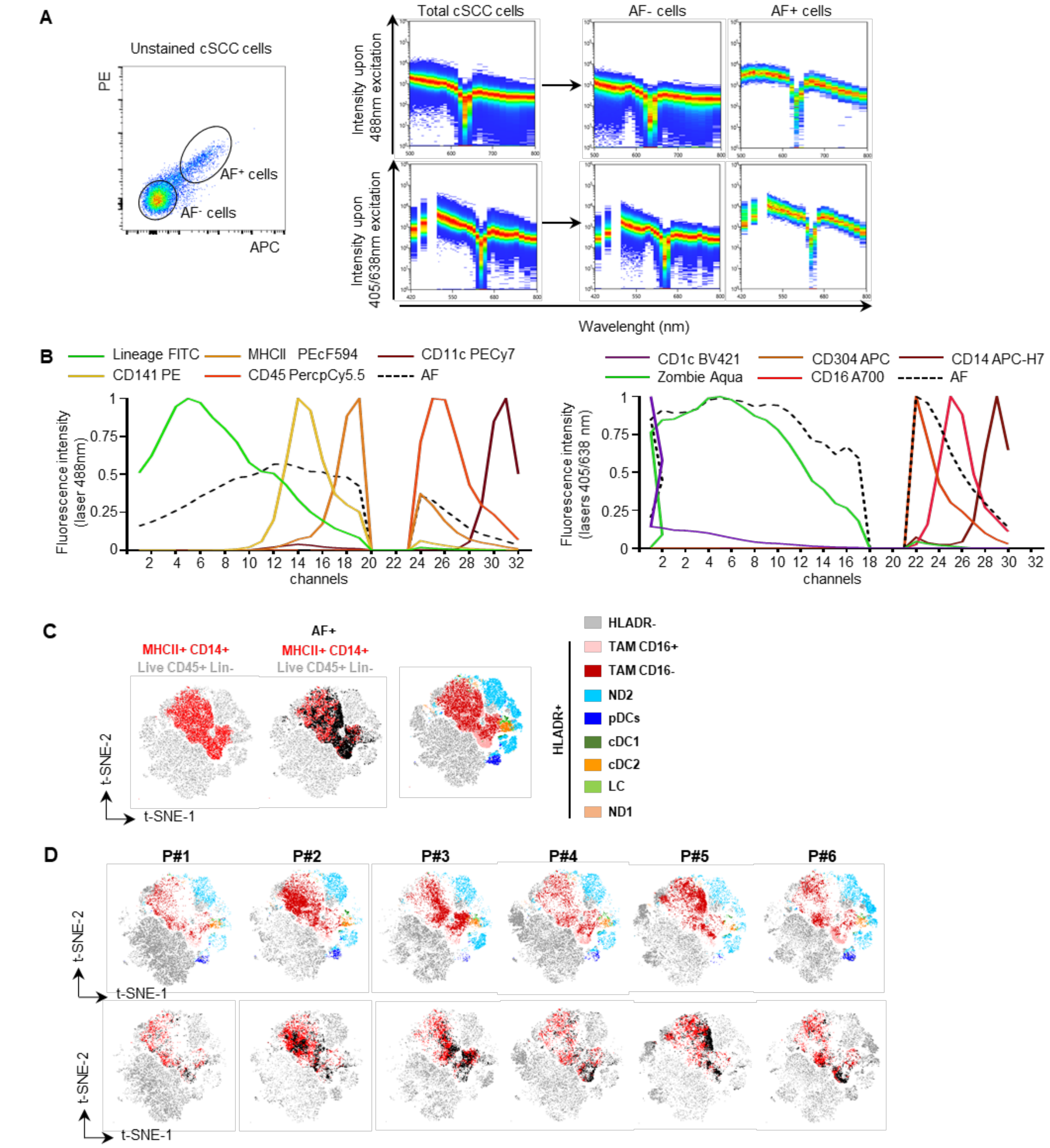

Supplementary Figure 1 : Autofluorescent macrophages infiltrate human cutaneous squamous cell carcinoma. (**A**) Bidimensional plot (left) showing the autofluorescent cells associated to unstained human cSCC before linear unmixing of raw data and corresponding emission *spectra* of total cells, autofluorescent and non autofluorescent cells after autofluorescence exclusion. (**B**) Emission *spectra* of the fluorescent dyes and autofluorescence. **(C-D)** tSNE plots on live $CD45^+Lin^-$ cells for six concatenated tumors (C) and for corresponding individual tumors (**D**) showing the cell clustering corresponding to the myeloid subsets identified in Figure 1A and autofluorescent cells that overlap with the $MHCII^+CD14^+$ TAM cluster (black dots).

Supplementary Figure 2 Bourdely et al.

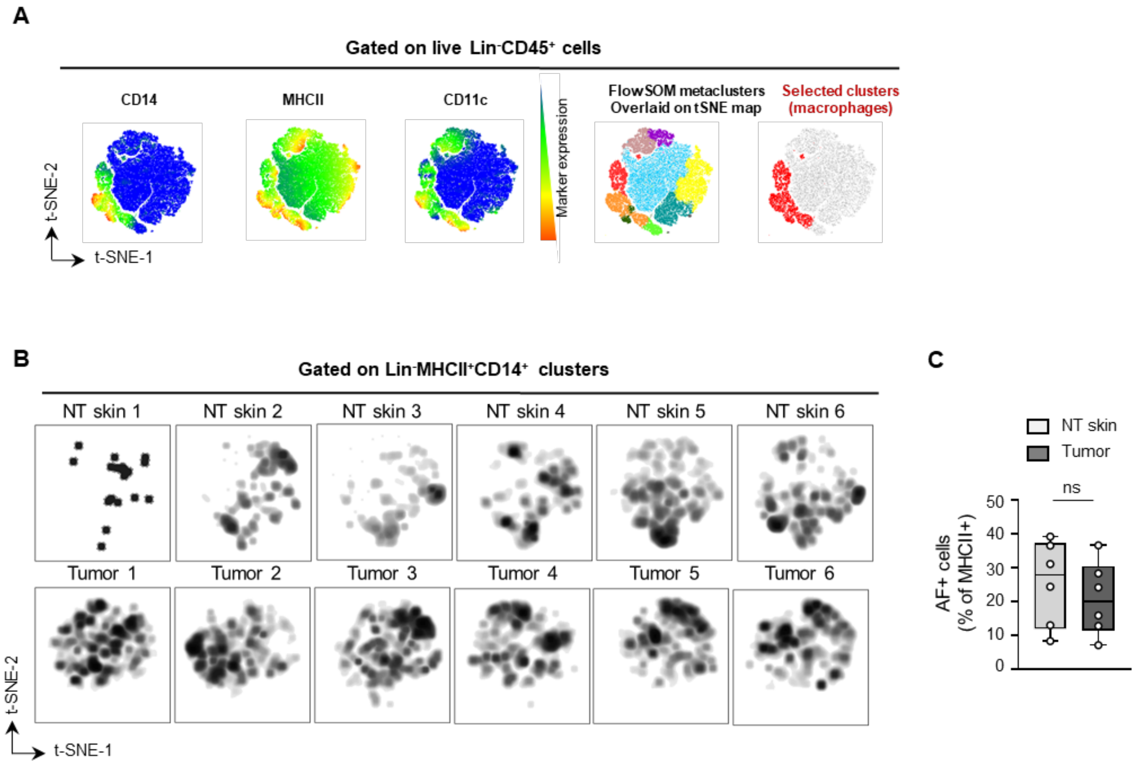

Supplementary Figure 2 : Identification of CD14$^+$MHCII$^+$ macrophages in human skin and cutaneous SCC. Unsupervised analysis of spectral flow cytometry data from human cutaneous squamous cell carcinoma (n=6) and non tumoral (NT) skin (n=6) samples on live on lin$^-$(CD3$^-$, CD19$^-$, CD15$^-$, CD56$^-$) CD45+ cells using CD14, MHCII, CD11c, CD206, CD204, CXCR4 and PD-L1 markers as well as the cell autofluorescence (AF). (**A**) t-SNE dimensional reduction on live Lin$^-$CD45$^+$cells using CD14, MHCII and CD11c markers. Live Lin$^-$CD45$^+$ subsets separated into 10 meta-clusters (MC) by FlowSOM automatic clustering were overlaid on the tSNE map. CD14$^+$ and MHCII$^+$ clusters corresponding to macrophages are shown in red on the tSNE map. (**B**) t-SNE dimensional reduction on live CD45$^+$Lin$^-$MHCII$^+$CD14$^+$ Macrophage subsets using CD14, MHCII, CD11c, CD206, CD204, CXCR4 and PD-L1 markers. Individual density plots of the tSNE map of live CD45$^+$Lin$^-$MHCII$^+$CD14$^+$ selected clusters represented in Figure 2A (500 events). (**C**) Frequency of AF+ macrophages among MHCII+ cells for each non tumoral and tumoral sample. (**D - see next page**) Macrophages subsets were separated into 45 meta-clusters (MC) by FlowSOM automatic clustering. Cells of each MC group overlaid on a t-SNE-1/t-SNE-2 map and their cell abundance represented as the cell proportion among live CD45$^+$Lin$^-$MHCII$^+$CD14$^+$ cells for each non-tumoral and tumoral sample. Mann-Whitney *U* test,* p<0.05, ** p<0.01, ns: not statistical.

Supplementary Figure 2D Bourdely et al.

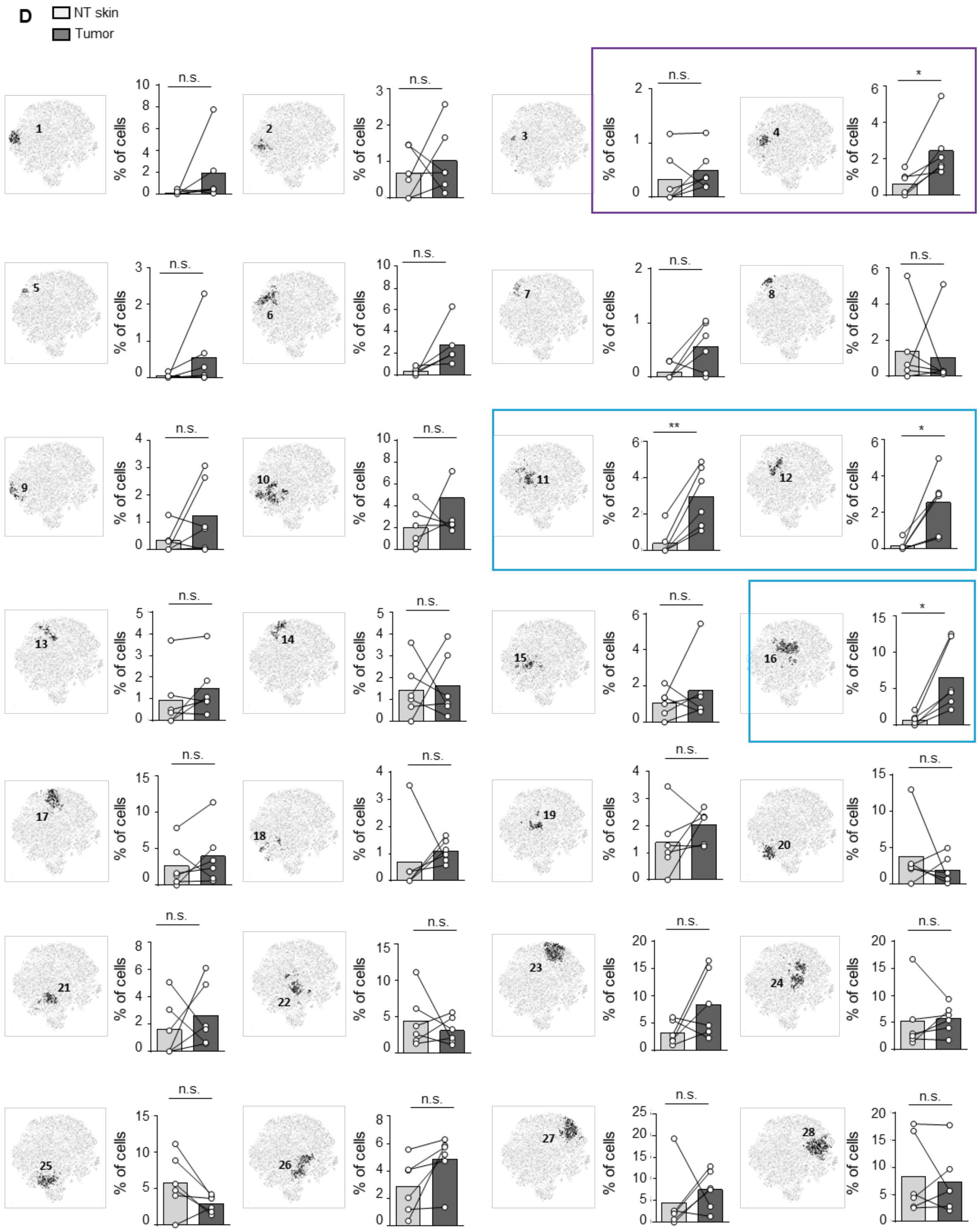

Supplementary Figure 2D Bourdely et al.

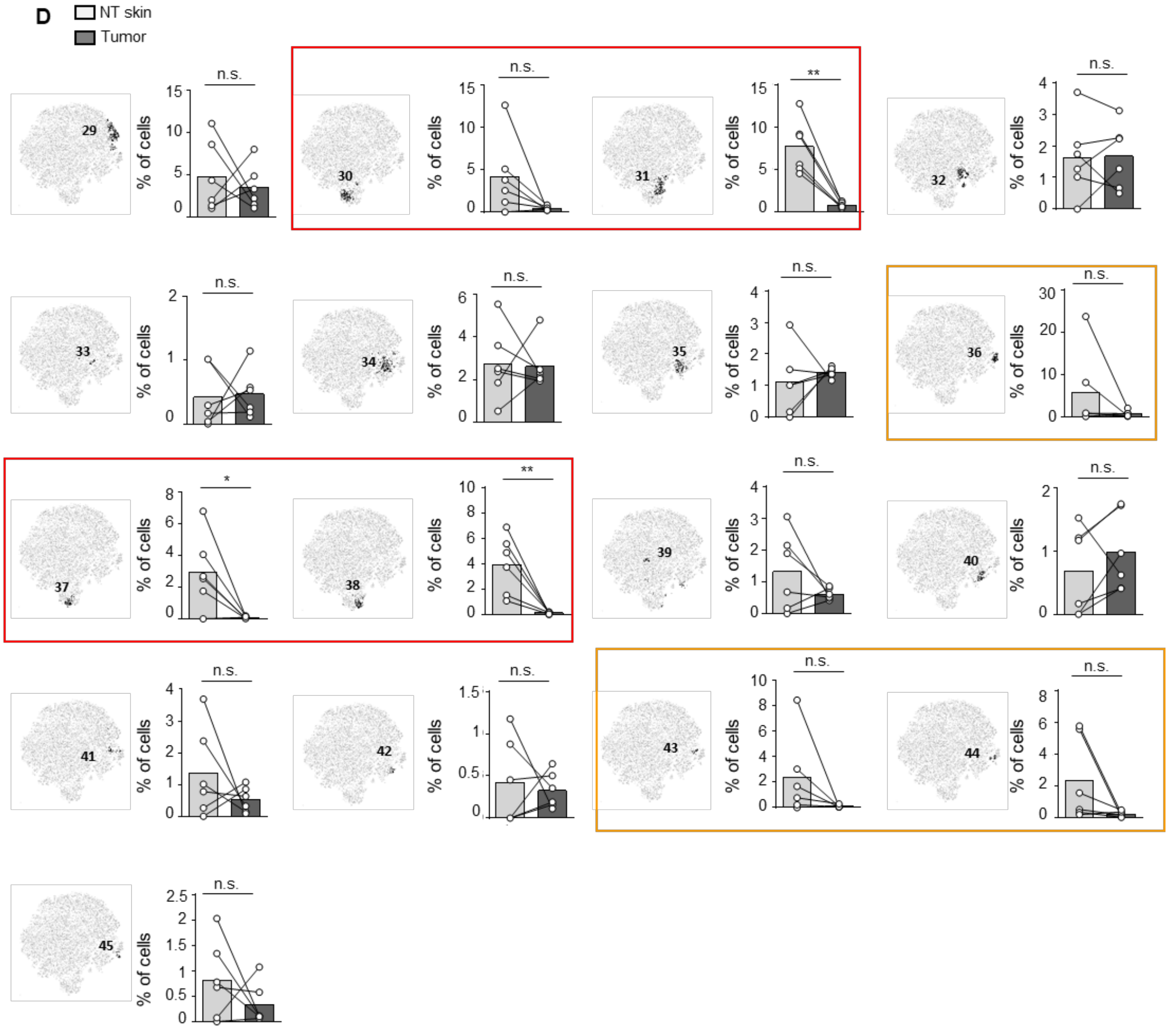

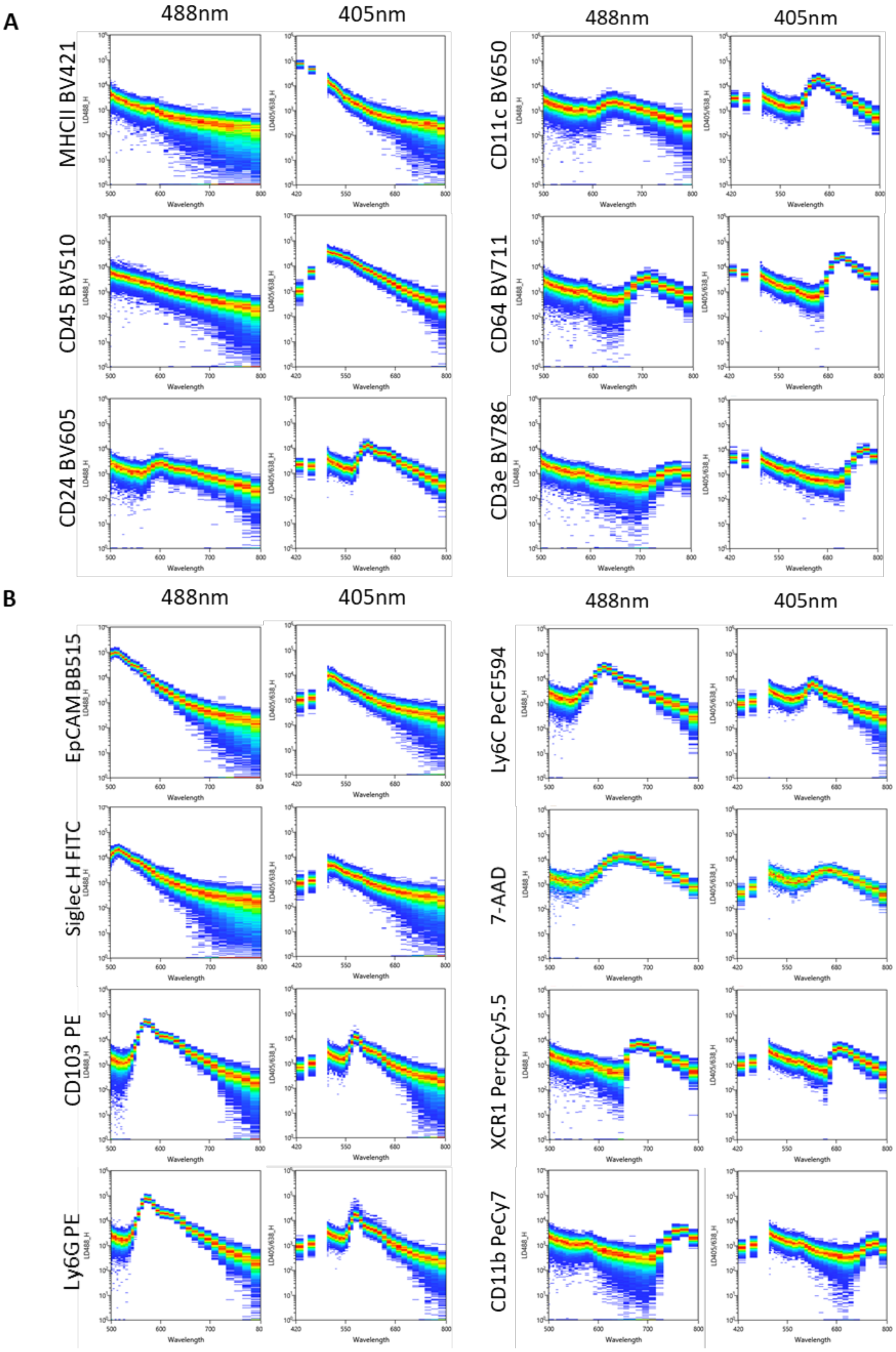

Supplementary Figure 3 : (A-B) Individual emission *spectra* of the fluorescent dyes used.

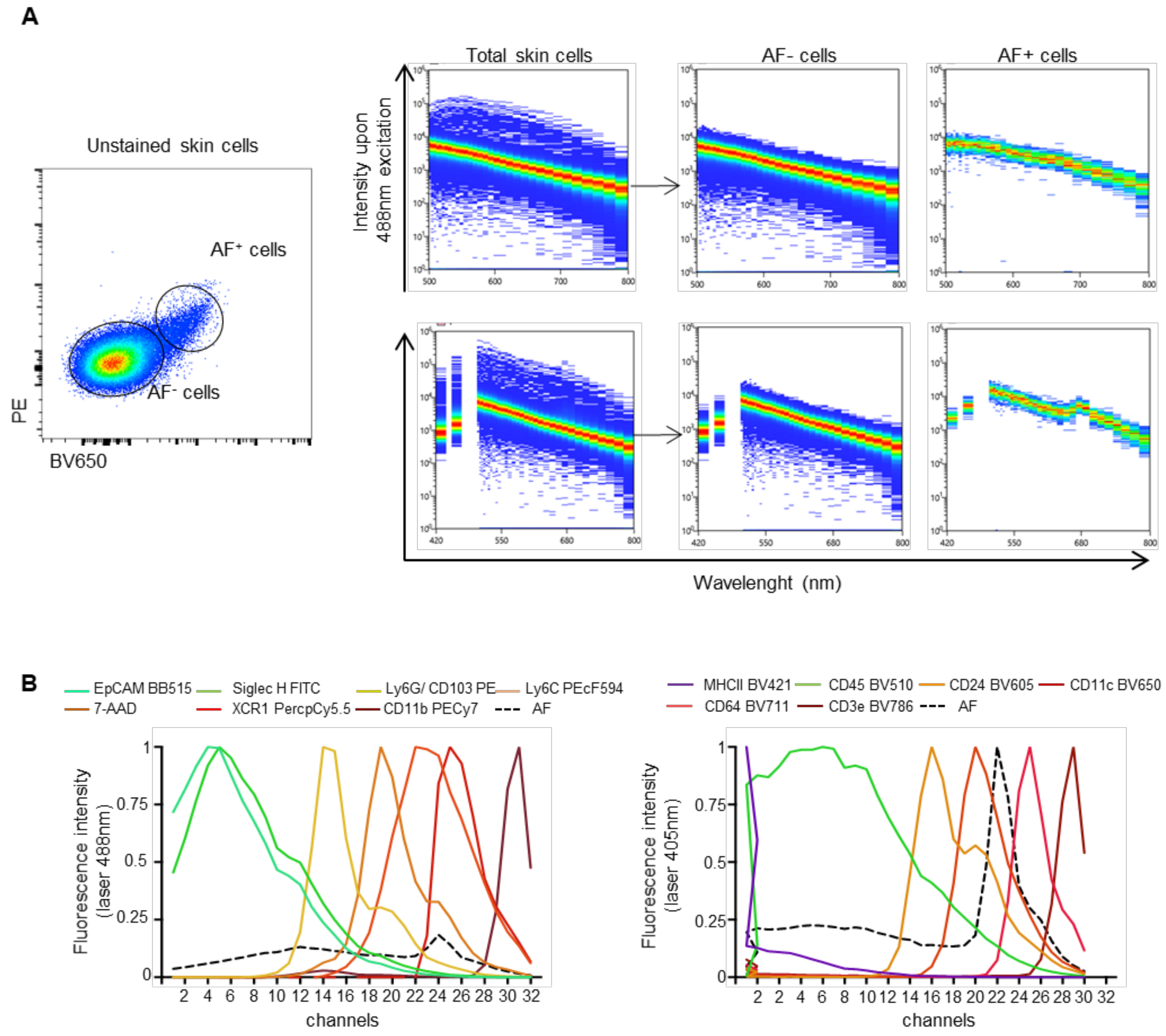

Supplementary Figure 4 : (**A)** Bidimensional pseudocolor plot showing the autofluorescence associated to unstained perilesional skins from 12-days TC-1 intradermal tumor-bearing mice (left) and corresponding emission *spectra* of total cells, autofluorescent and non autofluorescent cells after autofluorescence exclusion (right). (**B**) Emission *spectra* of the fluorescent dyes and autofluorescence.

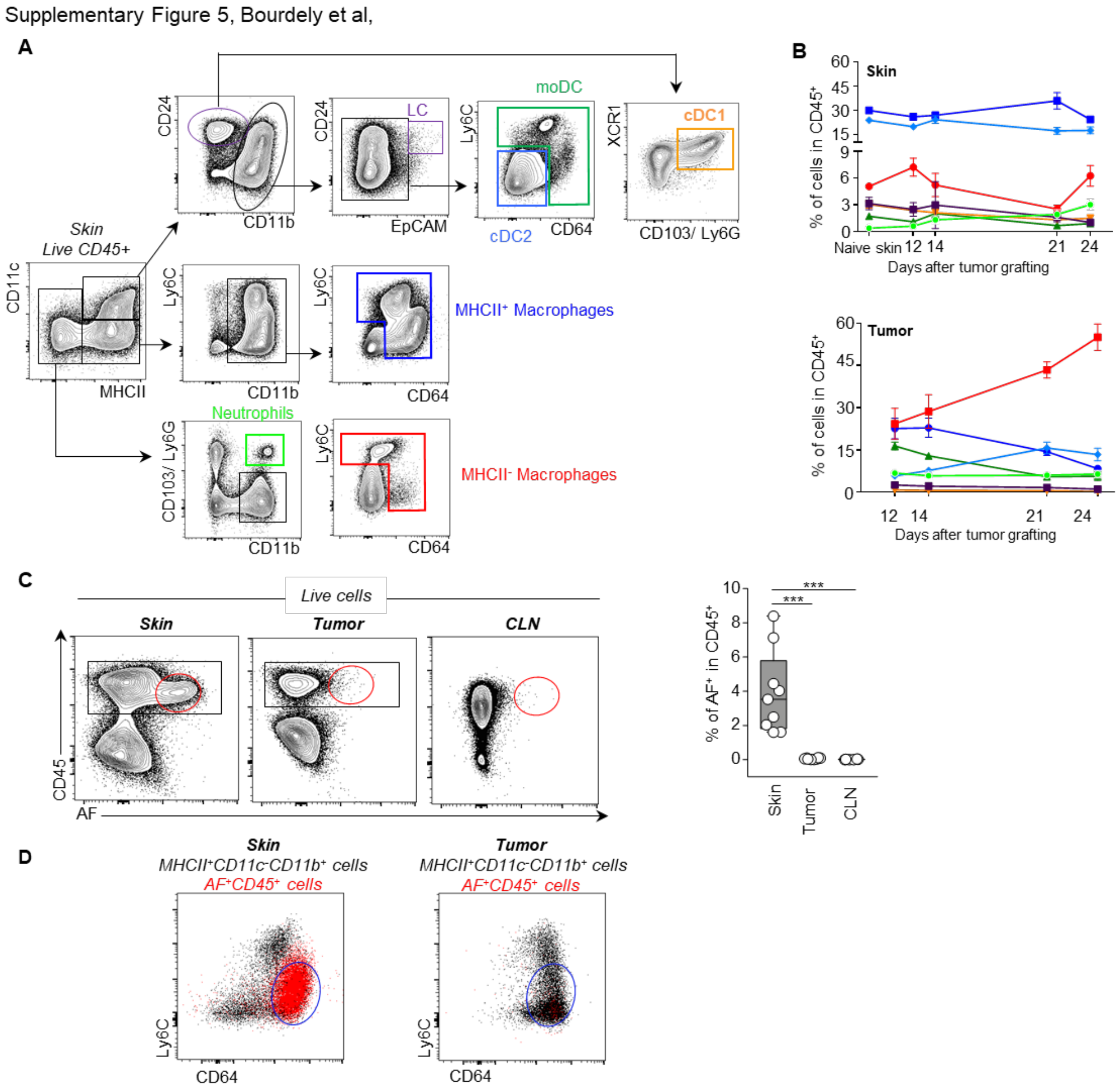

Supplementary Figure 5 : Characterization of the immune infiltrate of mouse skin and intradermal TC-1 tumors. (**A**) Representative flow cytometry plots in the perilesional skins from 12-days TC-1 intradermal tumor-bearing mice. (**B**) Frequencies of DC subsets, macrophages and neutrophils infiltrating TC-1 perilesional skins and tumors at the indicated time points upon intradermal TC-1 tumor cell line grafting. Values represent the mean±SEM from 3 independent experiments, n=5-9 per time point. (**C**) Flow cytometry plots and quantification of autofluorescent cells on live single cell suspensions from perilesional skin, 14-day cutaneous TC1 tumor and cutaneous draining lymph nodes (cLN) after multiparametric staining (for antibody panel see supplementary table X) showing autofluorescence associated to $CD45^{+}$ immune cells, ordinary one-way ANOVA, *** p<0.001. (**D**) Flow cytometry dot plots showing that autofluorescence (red dots) identified within live $CD45^{+}$ cells as shown in **C** overlaps with $MHCII^{+}$ macrophages (blue gate) in skin and tumor.

Supplementary Figure 6, Bourdely et al,

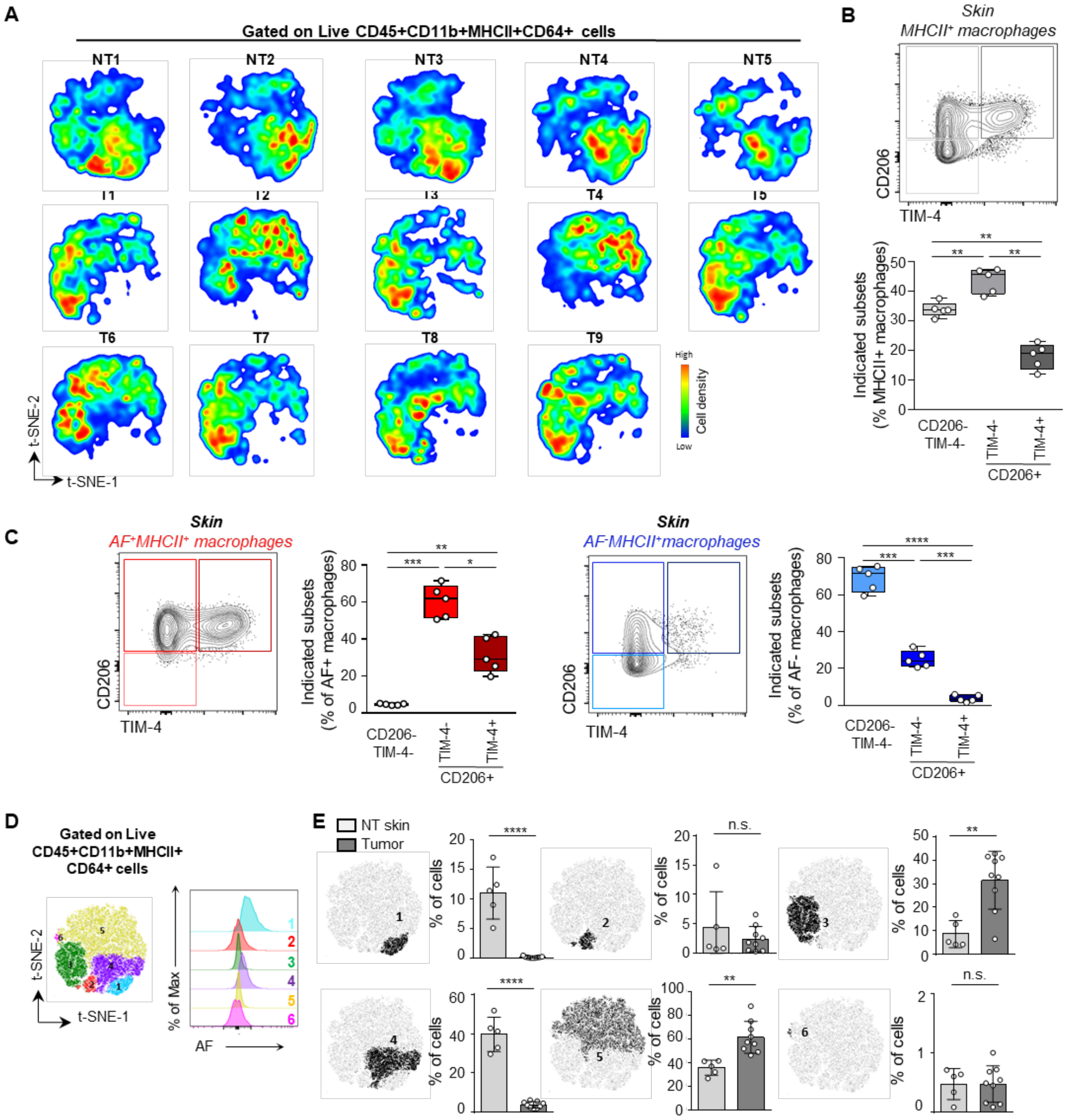

Supplementary Figure 6 : Characterization of MHCII$^+$ macrophage subsets based on autofluorescence in mouse skin and TC-1 intradermal tumor. Unsupervised analysis of spectral flow cytometry data from tumors (n=9) and non tumoral (NT) skin (n=5 pools of skin) of TC-1 intradermal tumor-bearing mice. (**A**) Individual pseudocolor density plots of the tSNE map of live CD45$^+$CD11b$^+$MHCII$^+$CD64$^+$ cells represented in Figure 3B for non tumoral (NT) and tumor samples. (**B-C**) Flow cytometry contour plots showing CD206 and TIM-4 expression in total MHCII$^+$ macrophages (**B**) and in AF$^+$ MHCII$^+$ macrophages (**C, left panel)** or in AF$^-$ MHCII$^+$ macrophages (**C, right panel**) for a representative skin and frequencies of each subset in all skin samples, n=5, paired T test, ** p<0.01. (**D**) Macrophages subsets were separated into 6 meta-clusters (MC) by FlowSOM automatic clustering. Colored clusters represented on the t-SNE map and flow cytometry histogram showing the AF intensity for each cluster. (**E**) Cells of each MC group overlaid on a t-SNE-1/t-SNE-2 map and their cell abundance represented as the cell proportion among live CD45$^+$CD11b$^+$MHCII$^+$CD64$^+$ cells for each non-tumoral and tumoral sample. Mann-Whitney *U* test,** p<0.01 and **** p<0.0001, ns: not statistical.

Supplementary Figure 7, Bourdely et al,

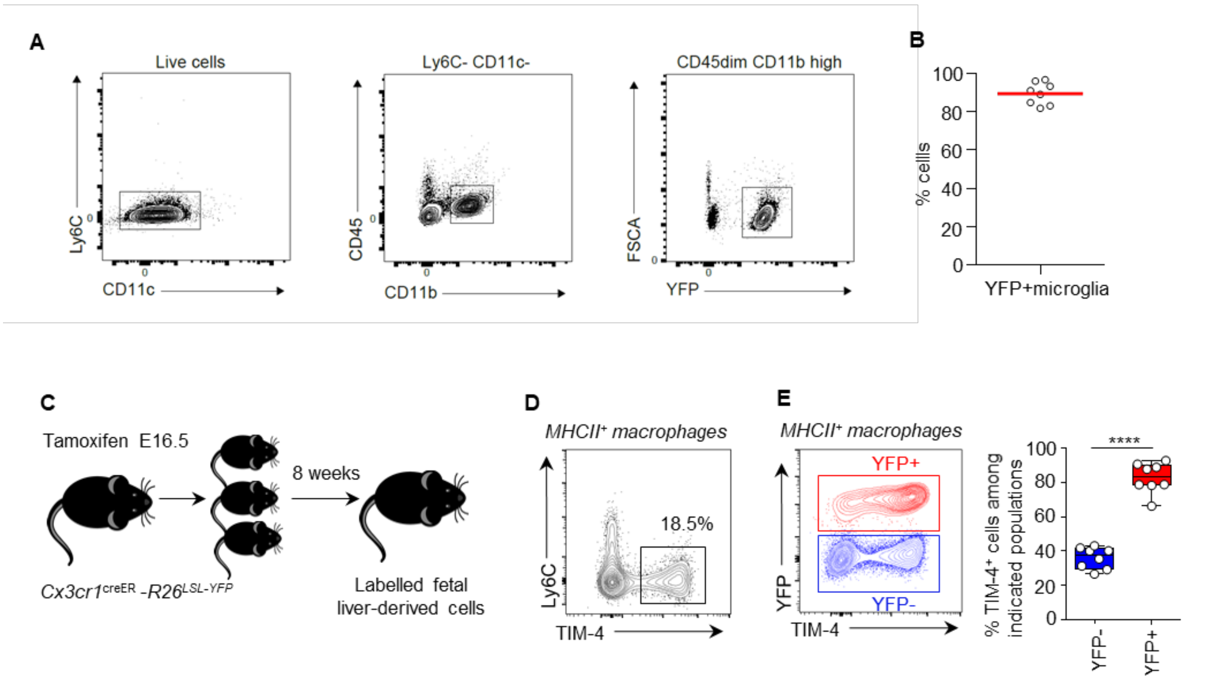

Supplementary Figure 7 : Fate mapping of of the skin MHCII$^+$ macrophages.

(A-B) Characterization of YFP$^+$ microglia from pulse-labeled CX3CR1CreER.R26-yfp mice. (**A**) Flow cytometry gating strategy showing YFP$^+$ microglia cells as CD45^{dim}CD11b^{hi} cells in LY6C$^-$CD11c$^-$ cells. (**B**) frequency of YFP$^+$ microglia. n=8. (**C**) Experimental model for the fate mapping of the skin macrophages by pulse-labelling of *Cx3cr1-R26*^{YFP} with tamoxifen at embryos E16.5. (**D**) Representative flow cytometry plot showing the abundance of TIM-4$^+$ macrophages among MHCII$^+$ macrophages in pulse-labeled CX3CR1CreER.R26-yfp mice. (**E**) Flow cytometry analysis showing the abundance of TIM-4$^+$ macrophages among YFP$^+$ and YFP$^-$ MHCII$^+$ macrophages, respectively, from the skin of CX3CR1-cre^{ert}YFP mice following tamoxifen i.p. Injection. Results come from two independent experiments (n=8). **** p<0.0001, paired t-test.

Supplementary Figure 8, Bourdely et al,

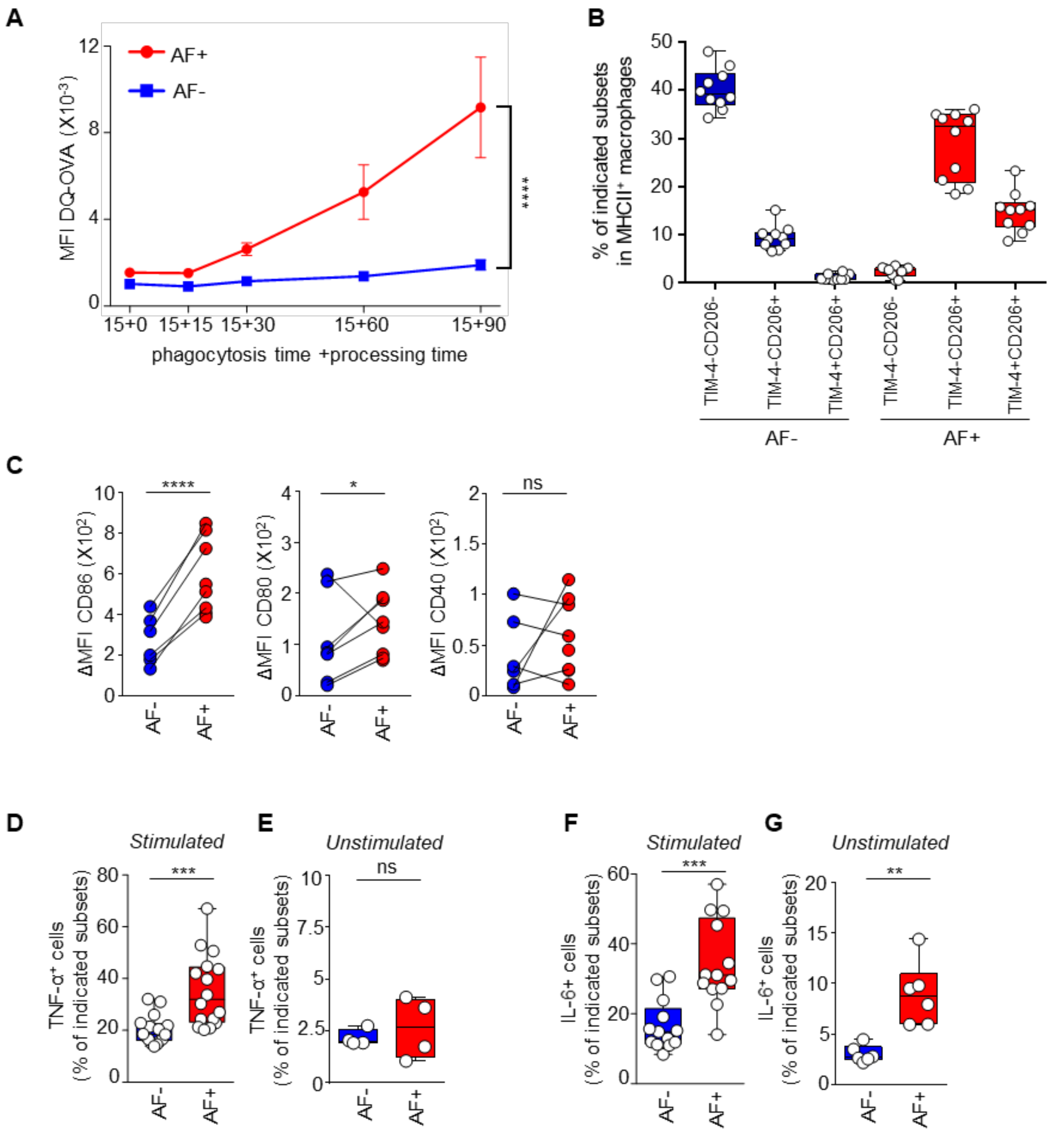

Supplementary Figure 8: Functional properties of autofluorescent macrophages.
(**A**) Quantification of *in vitro* uptake and processing of the quenched fluorescent dye DQ-OVA by skin AF$^+$ and AF$^-$ MHCII$^+$ macrophages in total skin cell suspensions for 15 min at 37°C (15+0) and follow up of the processing of the internalized dye at the indicated time points. Processing of DQ-OVA by macrophages was analyzed by flow cytometry after multiparametric staining (for antibody panel see supplementary Table 4). Results are expressed as median fluorescent intensities (corresponding to processing) shown for each time point in AF$^+$ and AF$^-$ MHCII$^+$ macrophages (n=7-10). p <0.0001, Two-way ANOVA. (**B**) Abundance of the indicated macrophage subsets among MHCII$^+$ macrophages in pHRodo experiment shown in Figure 5B. (**C**) Expression of CD86, CD80 and CD40 maturation markers upon LPS *in vitro* stimulation. (n=7), Mann-Whitney *U* test, **** p<0.0001. Graphs showing the ΔMFI CD86, CD80 and CD40 corresponding to the MFI upon LPS stimulation minus the MFI without stimulation, n=7, paired T test, * p<0.05 and **** p<0.0001. (**D-G**) Production of TNF-α (E-F) and IL-6 (G-H) by AF$^+$ and AF$^-$ mouse skin MHCII$^+$ macrophages analysed by flow cytometry in total skin cell suspensions upon in *vitro* stimulation with LPS (**D**) or ODN CpG2395 (**F**) or no stimulation (**E, G**). (**D-G**) Statistical analysis, Mann-Whitney *U* test, **p <0.01 and ***p <0.001.

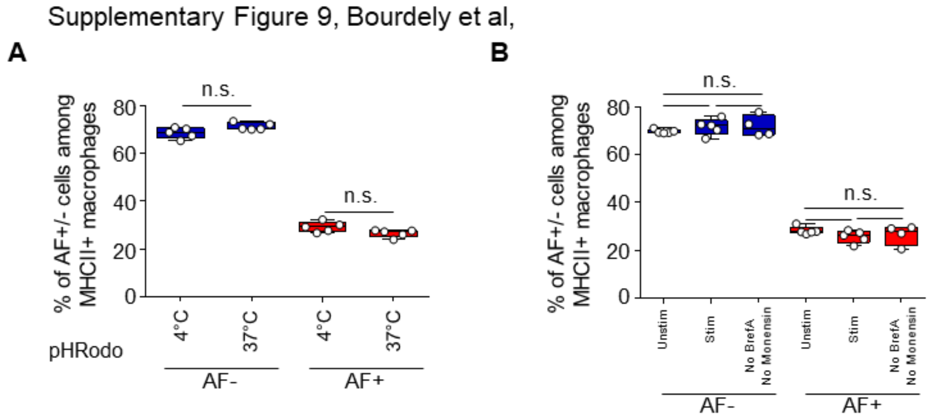

Supplementary Figure 9: the abundance of autofluorescent macrophages is not altered by in vitro treatments.

(**A**) Quantification of abundance of AF^+ and AF^- populations among $MHCII^+$ macrophages in mouse NT skin cell suspension incubated with pHrodo Green *E.Coli* BioParticle conjugates at 4°C and 37°C during 15 minutes. Statistical analysis, Mann-Whitney *U* test, n.s. p>0.05. (**B**) Quantification of abundance of AF^+ and AF^- populations among MHCII+ macrophages in mouse NT skin cell suspension after 4 hours incubation before intracellular cytokine staining without TLR stimulation (Unstim condition), upon stimulation with LPS at 37°C during 4h with GolgiStop and GolgiPlug (Stim condition) and upon stimulation with LPS at 37°C during 4h without GolgiStop and GolgiPlug (No BrefeldinA/NoMonensin condition). Statistical analysis, Mann-Whitney *U* test, n.s. p>0.05.
